# Identifying subgroup markers in heterogeneous populations

**DOI:** 10.1093/nar/gkt845

**Published:** 2013-09-20

**Authors:** Jorma J. de Ronde, Guillem Rigaill, Sven Rottenberg, Sjoerd Rodenhuis, Lodewyk F. A. Wessels

**Affiliations:** ^1^Division of Molecular Carcinogenesis, The Netherlands Cancer Institute, 1066 CX, Amsterdam, The Netherlands, ^2^Division of Molecular Biology, The Netherlands Cancer Institute, 1066 CX, Amsterdam, The Netherlands, ^3^Division of Medical Oncology, The Netherlands Cancer Institute, 1066 CX, Amsterdam, The Netherlands and ^4^Faculty of EEMCS, Delft University of Technology, 2628 CN, Delft, The Netherlands

## Abstract

Traditional methods that aim to identify biomarkers that distinguish between two groups, like Significance Analysis of Microarrays or the *t*-test, perform optimally when such biomarkers show homogeneous behavior within each group and differential behavior between the groups. However, in many applications, this is not the case. Instead, a subgroup of samples in one group shows differential behavior with respect to all other samples. To successfully detect markers showing such imbalanced patterns of differential signal, a different approach is required. We propose a novel method, specifically designed for the Detection of Imbalanced Differential Signal (DIDS). We use an artificial dataset and a human breast cancer dataset to measure its performance and compare it with three traditional methods and four approaches that take imbalanced signal into account. Supported by extensive experimental results, we show that DIDS outperforms all other approaches in terms of power and positive predictive value. In a mouse breast cancer dataset, DIDS is the only approach that detects a functionally validated marker of chemotherapy resistance. DIDS can be applied to any continuous value data, including gene expression data, and in any context where imbalanced differential signal is manifested.

## INTRODUCTION

**Figure 1.**
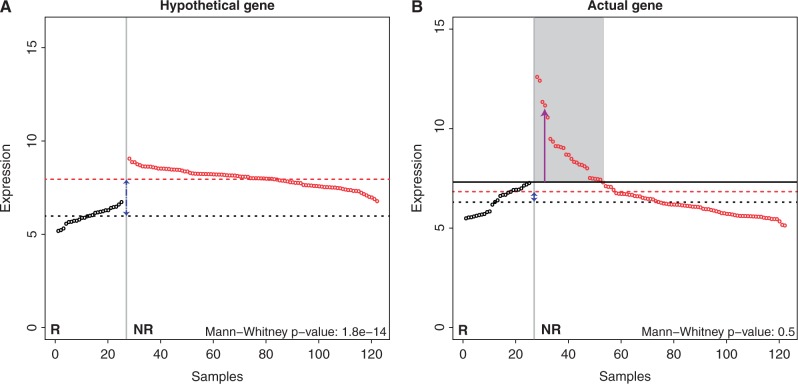
Gene expression patterns of genes suitable for group-wise analyses and DIDS. (**A**) Expression levels across controls and cases for a hypothetical gene suitable for group-wise analysis. A conventional approach using, e.g. a Mann–Whitney test, would easily identify this gene. The black symbols represent samples in the control group and the red symbols the samples in the case group. The gene expression values have been sorted from low to high for the control group and from high to low for the case group. The dashed black (red) line represents the average gene expression in the control (case) group. (**B**) The expression levels of an actual gene we identified using DIDS. The maximum expression level in the control group is indicated by the solid black line. The bulk of the cases show expression levels similar to the control samples; however, a subgroup exists that shows expression levels that are above the maximum in the control group and clearly deviating from the expression levels in that group (shaded region). DIDS is designed to specifically detect these patterns.

For the identification of biomarkers, genome-wide microarray gene expression analyses have, so far, mostly been focused on group-versus-group comparisons. For example, to detect markers of therapy resistance, one would compare mRNA expression profiles of tumors that are sensitive to chemotherapy with the profiles of tumors that are resistant to therapy. Unfortunately, this approach has been unsuccessful in identifying markers of chemotherapy resistance (1,2). Traditional methods for group-versus-group comparisons, like the *t*-test, Mann–Whitney test or the Significance Analysis of Microarrays (SAM) approach (3), rely on comparing the expression of a candidate gene in the responder group with the expression of this gene in the non-responder group. This approach is mostly suited for tumors that show highly similar expression levels within a group, while the average expression levels between the groups are dissimilar. This implies homogeneous expression within a group. Figure 1A depicts the gene expression of a hypothetical marker gene showing homogenous expression levels within each group. Approaches like the Mann–Whitney test would be the method of choice for detecting such markers. For this gene, the *P*-value associated with the Mann–Whitney test is highly significant (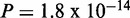). However, when expression is heterogeneous within one of the two groups, these methods are underpowered to detect this type of differential signal.

Heterogeneous expression within a group could arise in many situations. For example, in breast cancer, response to chemotherapy is highly heterogeneous, even within a molecular subtype (4). This is most likely due to the large array of mechanisms that can cause resistance to therapy (5). It has recently become clear that even when a resistance mechanism is identified, only a minority of the resistant tumors might harbor that particular mechanism (6). It follows that a marker of such a mechanism will only show aberrant expression in a relatively small subgroup of the non-responder group, while the majority of the non-responders will show expression similar to the responders.

Figure 1B depicts a gene that only shows aberrant expression in a subset of the non-responder group. Consequently, the median expression of the responders is not significantly different from the median expression in the non-responder group (Mann–Whitney test, 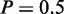). However, as indicated in Figure 1B, a subset of the non-responder samples displays gene expression levels significantly higher than the highest levels in the responder group (shaded region). This indicates that this subset behaves differently compared with the other cases, and this gene could be a marker of resistance to therapy present in only this subset.

More generally, when comparing cases (such as non-responders) with controls (such as responders) and when we aim to discover mechanisms that are present in a small subset of the case group, we need to apply an approach tailored to this situation. A number of methods have already been proposed that take such heterogeneity within the cases into account. These include approaches such as Cancer Outlier Profile Analysis (COPA) (7,8), Outlier Sums (OS) (9), Outlier Robust T-statistic (ORT) (10) and Maximum-Ordered Subset T-statistics (MOST) (11). Also, the two-sample Kolmogorov–Smirnov (KS) test could be considered, as it aims to identify differences between two distributions, not just a shift in mean.

Existing approaches have several drawbacks that make them less suited to the problem of supervised subgroup marker detection. First, COPA and OS base their threshold for outlier detection on all samples, i.e. these approaches do not exploit knowledge of the levels of expression in one group (controls) to optimize detection of aberrant signal in the other group (cases). Although ORT and MOST do base their outlier definition on samples in the control group, these methods do not constrain the aberrant signal to exceed expression levels of ‘all’ samples in the control group. Although setting the threshold to the maximum of the control group leads to more stringent candidate selection, we are looking for markers that offer clear differences between cases and controls. In case some controls also show aberrant expression, the interpretation of the role this marker plays in the phenotype under investigation will become more complex, which, in turn, will lead to more complex follow-up experimentation. We chose to optimize for clear-cut expression patterns and high positive predictive value (PPV) in the candidate marker list to increase the success rate of follow-up experiments. Second, none of these methods offer an exact, permutation-free (and therefore much faster) *P*-value calculation. Finally, none of these approaches allow for the detection of specific, defined patterns of aberrant signal. A specific pattern could, for example, be the highly aberrant expression of a small subset of samples as opposed to the moderately aberrant expression of a larger subset. In the first scenario, a scoring function could be used that assigns high scores to genes highly aberrantly expressed in a small subset of samples (e.g. a quadratic scoring function), whereas the second scenario would require a scoring function that attenuates high expression values (e.g. the tanh function). Here we propose a novel method that addresses all these shortcomings. Extensive comparisons on artificial and real datasets confirm its superior performance.

## MATERIALS AND METHODS

### Datasets

To compare the approaches, we used an artificial synthetic dataset (artificial dataset), a semi-artificial dataset generated from gene expression data from selected breast cancer samples (HER2 dataset) and a mouse dataset for which a functionally validated gene implicated in chemotherapy resistance is known (mouse dataset). Here we provide detailed descriptions of these datasets.

#### Artificial dataset

The first, artificial, gene expression dataset was designed to mimic a situation where a group of controls is compared with a group of cases where only a (small) subgroup of the cases show aberrant expression. Such a set is ideal to compare the performance of various methods and different scoring functions under a variety of circumstances, as the identity of the true marker genes is known. For these datasets, we generated two types of genes: (i) reporter genes that do show differential expression between the control and case groups and (ii) non-reporter genes that do not show differential expression between controls and cases. We constructed datasets containing 25 000 genes, of which 250 (1%) are reporter genes. For reporter genes, the expression values for samples in the control group were sampled from a unit variance, zero mean, normal distribution denoted by 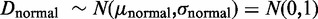. For the case group, the expression values of a percentage (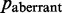) of the samples were sampled from 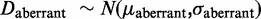, with 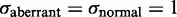 and 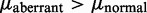. The expression values for the remaining (i.e. 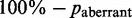) case samples were sampled from 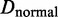. For non-reporter genes, the expression levels of all samples (both controls and cases) were sampled from 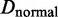. By varying the percentage of aberrant samples, i.e. 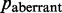, and the difference between the two means (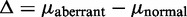), we simulated a number of different conditions.

For the power calculations, we only simulated reporter genes (positives) and calculated *P*-values by constructing an empirical null distribution for each method. We did so by generating 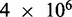 non-reporter genes (negatives), computing the score for each method for all these non-reporter genes and determining the relevant quantiles of the observed scores.

We simulated three different scenarios, each characterized by a specific combination of the number of samples in the control (*n*_1_) and case (*n*_2_) groups. The scenarios represent a balanced dataset and two datasets where the group sizes are unbalanced. These scenarios are (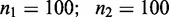), (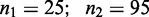) and (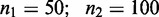). We chose these scenarios, as they reflect class sizes and ratios that we frequently encounter in patient series. Finally, we included a scenario where only a limited number of samples are available (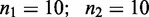). Because this will result in a very low absolute number of aberrant cases, we were interested in seeing how well Detection of Imbalanced Differential Signal (DIDS) would perform under these circumstances.

#### HER2 dataset

We used the second dataset to assess the performance of the approaches under simulated real-life conditions where a positive control is present. For this purpose, we selected, based on immunohistochemistry, 178 HER2-negative samples from a breast cancer dataset, of which 98 samples represented the complete control group and 80 samples the non-aberrant samples in the case group (GEO accession ID GSE34138). Next we randomly selected 10 HER2-positive tumors to constitute the samples in the case group showing aberrant gene expression (GEO accession ID GSE41656). We repeated this procedure 1000 times, during each repeat randomly selecting HER2-positive and HER2-negative samples as described above. By doing so, we generated 1000 datasets, each consisting of 188 samples and 27 506 probes. The HER2-positive tumors typically show aberrant expression for the genes on the HER2 amplicon. In fact, the positive control genes were defined as all genes residing on 17q12 or 17q21.1 (also see Supplementary Figure S1 for the genomic location of these genes). Therefore, each of the 1000 datasets has the characteristic that for the positive control genes (genes residing on the HER2 amplicon) a fraction (10 of 90) of the case group can show aberrant gene expression (upregulation). Note that because the grouping was based purely on the HER2 immunohistochemistry, it is possible that a sample shows positive HER2 staining, but no, or very low, over-expression of the HER2 mRNA. In fact, false-positive rates of up to 20% have been reported for HER2 staining (12). While we believe the false-positive rate for our dataset to be significantly lower owing to careful revision of an experienced breast pathologist, this dataset represents an excellent example of the noisy nature of real biological datasets. Because only a small fraction (10 of 90) of the case group consists of these HER2-positive tumors, the positive control genes will most likely not be picked up by a group-versus-group analysis (like a Mann–Whitney test or the SAM procedure).

#### Mouse dataset

Finally, we used the data derived from a set of mouse tumors that arose spontaneously in the 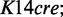 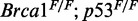 mouse model, a genetically engineered mouse model for breast cancer. The tumors that we used were treated with docetaxel, and in 21 cases, the tumors responded well to the treatment, whereas 22 tumors were resistant to the treatment. In five of the non-responding cases, poor response could be directly associated with a substantial upregulation of the mouse P-glycoprotein genes (ABCB1A and ABCB1B) (6). Each of the primary tumors from both the responder and non-responder group was subjected to genome-wide gene expression profiling. We ran the different analytical methods on this mouse dataset [i.e. 22 poor responder samples (cases) versus 21 docetaxel-sensitive tumor samples (controls)] and recorded at which positions the functionally validated positive control (ABCB1B) was found.

#### Tools

The *R* statistical programming language was used for all of the analyses described in this article.

### The DIDS algorithm

To detect genes (or other features) with aberrant signal in a small subgroup of cases, i.e. to detect genes that show ‘imbalanced differential signal’, we perform the following steps. For each gene, we calculate the maximum expression in the control group (solid black line in Figure 1B). Then we identify the outlier samples, i.e. the samples in the case group that exceed this threshold (samples indicated by the gray region in Figure 1B). For these outlier samples, we compute the ‘excess expression’, i.e. the amount by which the expression in the outlier samples exceeds the maximal expression in the control group. The contribution of each outlier sample to the DIDS score is computed by passing its excess expression through a scoring function. The final value of the DIDS score is obtained by summing all the contributions of the outlier samples. We implemented three scoring (also see Supplementary Figure S2). The type of scoring function determines the kind of aberrant expression patterns that can be detected. These include candidate genes where a relatively large number of case samples show a relatively small degree of excess expression or a relatively small number of case samples that show a relatively large degree of excess expression. Both approaches have their merit, as it has been shown that even small differences in gene expression can be linked to resistance to chemotherapy. On the other hand, larger excess expression values provide more confidence that the difference is not an artifact of technical origin. In what follows, we provide a formal description of the algorithm.

Let the gene expression values of the *n*_1_ control samples for a gene be given by

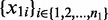

and the gene expression values of the *n*_2_ case samples for a gene be given by

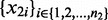



To detect genes that show higher expression in case samples compared with control samples, we first define the maximal gene expression value of a gene among the control samples as

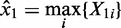



The DIDS score is then given by
(1)
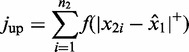

where

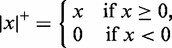



and *f* is a strictly increasing function. We use the following three variants of *f*:

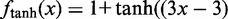


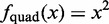


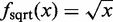



The scoring functions were chosen so that distinct types of aberrant gene expression patterns can be detected. See Supplementary Figure S2 for more details on the characteristics of the three scoring functions.

The procedure as outlined above is used to detect genes that show ‘increased’ expression in a subgroup of case samples. For detecting genes with ‘decreased’ expression, the procedure is analogous, except that a threshold is set at the ‘minimal’ expression level in the control group, and that cases with expression levels ‘lower’ than this threshold contribute to the DIDS score (also see Supplementary Materials and Methods).

To quantify the statistical confidence associated with a given value of the DIDS score, we compute a *P*-value for each gene. To this end, we developed an analytical procedure to compute permutation-based exact *P*-values. (See the Supplementary Methods for a detailed description of the procedure). This analytical procedure is very fast and results in more accurate *P*-values than the computational equivalent of performing the actual permutations, because it takes into account all possible permutations of the data. However, it should be stressed that permutation-based *P*-values, like our analytical procedure, only provide a *P*-value that reflects the likelihood of having the observed number of cases with an expression value above (below) the maximal (minimal) expression of the control samples. In other words, the *P*-value does not take the *magnitude* of the excess expression in the cases into account. As a consequence, the *P*-value we estimate is a conservative *P*-value.

Genes with a nominal *P*-value exceeding the user-defined α-level (set to 5% by default) are removed from the candidate list. The genes remaining after this filtering step are then ranked based on the DIDS score (for more details, the reader is referred to the Supplementary Methods and Supplementary Figure S3). The *R* implementation of the DIDS algorithm is freely available at http://bioinformatics.nki.nl/software.php.

### Power comparisons and PPV comparisons

Following earlier publications (9,10), we simulated a large number of samples under the null hypothesis (see ‘Datasets’ for details). Using these simulations we can estimate, for every statistic (or score), its distribution under the null. With this null distribution, we can then determine which value of the statistic (or score) corresponds to a specific false-positive rate (i.e. what fraction of the genes drawn from the null distribution is falsely called positive, also known as the α-level). This enables a fair comparison between the different methods, as we can compare their power at the same α-level (and not at α-levels that are influenced by the α-level estimation accuracy of each individual method). Subsequently, we varied the false-positive rate (α) and computed the corresponding power represented by the fraction of reporter genes (true positives) detected at the given false-positive rate. Given the power for each method in each scenario and for all parameter settings, we then computed, for each pair of methods, the difference in power as a function of α, 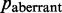 and Δ.

To evaluate the ability of the methods to identify a short, but pure, candidate list of reporters, we used the PPV, defined as the percentage of true positives in the top *N* candidate genes. Analogous to the power calculations on the artificial dataset, we again generated artificial datasets for the same three scenarios, varied 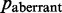 and Δ as before and computed the PPV in each case.

## RESULTS

### Method validation

To measure the performance of our method and to compare it with similar approaches, we used (i) an artificial synthetic dataset (artificial dataset); (ii) a dataset consisting of selected samples from a breast cancer patient series where the imbalanced signal is introduced by the presence or absence of HER2-positive tumors (HER2 dataset); and (iii) a mouse dataset for which a functionally validated gene implicated in chemotherapy resistance is known (mouse dataset; see ‘Materials and Methods’ section for a detailed description of the datasets). Each of these datasets was analyzed using DIDS, the SAM procedure, the Mann–Whitney test, the two-sample KS test, the *t*-test with equal variance (e.v.) and unequal variance (u.v.) and a selection of other algorithms that take heterogeneity within groups into account when detecting differential expression: MOST, OS, COPA and ORT.

Our validation experiments include both power comparisons and evaluations of the PPV. Power comparisons characterize methods in terms of the true-positive rate (sensitivity) that can be achieved at a fixed false-positive rate (1-specificity). Although power comparisons are a useful benchmark, we consider the PPV [the fraction of the called genes that are true positives (real markers)] to be a more relevant measure. This stems from the fact that we are using our approach to identify ‘candidate’ markers, which requires the called list to be as pure as possible to minimize the amount of resources spent on follow-up validations. In such a context, a short candidate list with a high percentage of true positives is preferred to a longer list with a lower percentage (but higher absolute number) of positives. Therefore, we strive for a maximal PPV rather than maximal power, although the two are clearly related.

### Results on the artificial dataset

For both the power and PPV comparisons, we generated instances of the artificial dataset representing three scenarios, each characterized by a specific combination of the number of samples in the control (*n*_1_) and case (*n*_2_) groups. The scenarios represent a balanced dataset and two datasets where the group sizes are unbalanced. These scenarios are (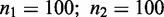), (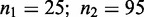) and (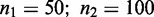). Finally, we included a scenario with a very small number of samples available: (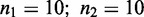). Within each scenario, we varied two parameters associated with the artificial dataset: (i) the percentage of samples in the case class showing aberrant expression, which was varied between 1 and 39%, i.e. 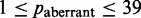 and (ii) the differences between the means of the distributions from which the aberrant and normal expression values were sampled (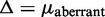 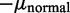), which were varied between 1 and 4, i.e. 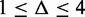, with 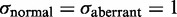 (See ‘Materials and Methods’ section for definitions of all parameters). Because the variance is the same in all scenarios, a change in delta corresponds to a change in the signal-to-noise ratio. Consequently, lower delta values correspond to noisier data, whereas higher delta values correspond to clearer signal.

#### Power comparisons

**Figure 2.**
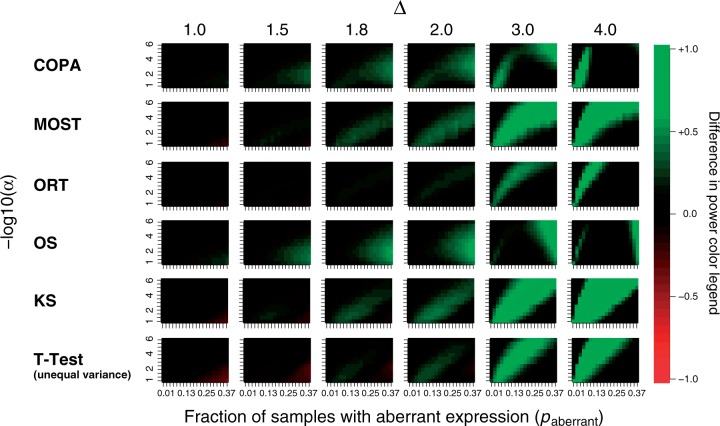
The difference in power between DIDS and other tested approaches for the scenario (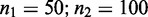). The differences are represented for all methods developed for imbalanced signals and the unequal variance *t*-test, as representative of conventional group-wise analyses. The methods are represented in the rows and the different values of Δ in the columns. For each combination of a method and a value of Δ, the power difference for DIDS and the method represented in the column are depicted as a function of 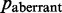 (horizontal axis) and α (vertical axis). Shades of green represent settings where DIDS has more power than the other methods, whereas shades of red represent settings where DIDS has less power than the other methods.

The results for all methods developed for imbalanced signals, the two-sample KS test (as a representative of tests that detect differences between distributions other than just the mean) and the unequal variance *t*-test (as representative of conventional group-wise analyses) are represented in Figure 2 for the scenario (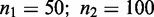). These results are representative for all other scenarios and all methods not depicted here. Full results for all methods and scenarios are depicted in Supplementary Figures S4–S8.

From Figure 2, it can clearly be seen that DIDS outperforms all other methods, except for the *t*-test in the scenario with relatively high percentages of aberrant samples (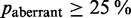), small differences (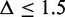) and large values of alpha (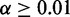). Given the large number of hypotheses that are typically evaluated (tens of thousands of genes) and the fact that usually only a limited number of candidates can be followed up, it follows that one would generally be especially interested in *P*-values that are well below this level. Consider the worst case scenario, where there are no genes with true signal. Then setting 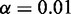 would correspond to a list of 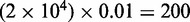 genes, on average, passing the α cutoff (i.e. all false positives). If we now consider a scenario where a percentage of the genes do show true signal, then 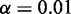 will result in >200 genes, as we expect genes with true signal to have lower *P*-values than genes with no signal. Given that only a 100 (or fewer) genes can typically be selected for follow-up experiments, it follows that we are interested in power comparisons at α levels well below 0.01. For all scenarios with *P* < 0.01, DIDS performs equal or better than any of the other algorithms.

#### PPV comparisons

**Figure 3.**
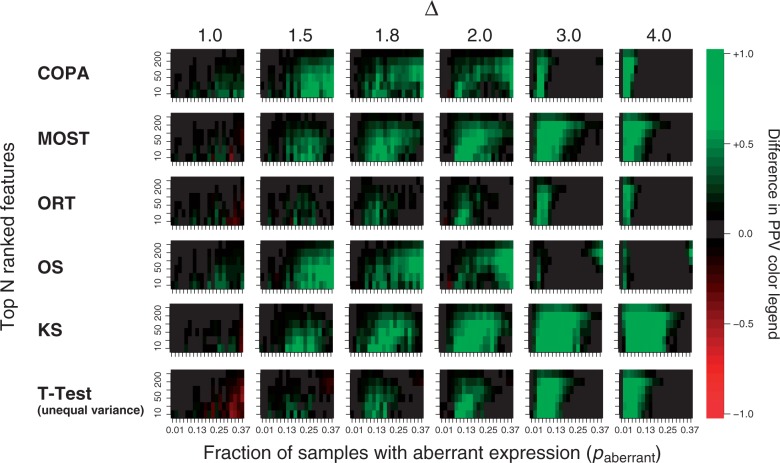
The difference in PPV between DIDS and other tested approaches for the scenario (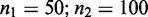). The differences are represented for all methods developed for imbalanced signals and the unequal variance *t*-test, as representative of conventional group-wise analyses. The methods are represented in the rows and the different values of Δ in the columns. For each combination of a method and a value of Δ, the differences in PPV for DIDS and the method represented in the column are depicted as a function of 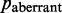 (horizontal axis) and the top *N* candidates (vertical axis). Shades of green represent settings where DIDS achieves a higher PPV than the other methods, and shades of red represent settings where DIDS achieves lower PPV than the other methods.

As for the results on power comparisons, results for all methods developed for imbalanced signals, the unequal variance *t*-test and the two-sample KS test are represented in Figure 3 for the scenario (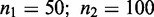). As before, these results are representative for the full results for all methods and scenarios as depicted in Supplementary Figures S9–S13.

Again, DIDS outperforms all other methods in virtually all scenarios. Only for relatively high percentages of aberrant samples (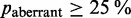) and a small difference (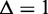), DIDS is outperformed by the *t*-test. Especially for 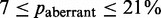 and 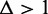, the advantage of DIDS over other methods is substantial, suggesting improved candidate list generation in real world analyses, characterized by a relatively small subset of samples displaying aberrant gene expression.

#### DIDS scoring functions

Rows 1 and 2 of Supplementary Figures S4–S13 show the direct comparison of 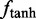 to 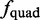 and 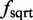, respectively. From these rows in these figures we observe that, as expected, the 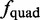 scoring function performs best for a small percentage of aberrant samples (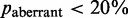) and for large values of Δ, i.e. when 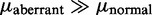.

### Results on the HER2 dataset

**Figure 4.**
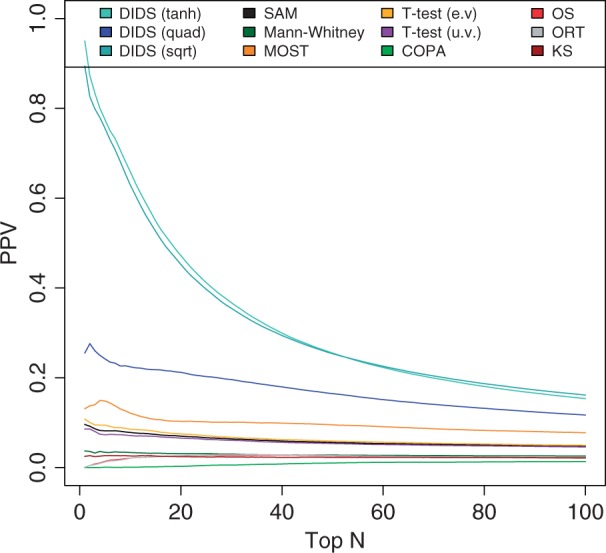
PPV comparison on HER2 dataset. PPVs for all algorithms on the HER2 dataset as a function of the top *N* candidates. The different variants of DIDS using the different scoring functions are denoted by ‘DIDS (tanh)’, ‘DIDS (quad)’ and ‘DIDS (sqrt)’, respectively.

The second artificial dataset consists of HER2 non-amplified samples admixed with HER2-amplified samples. In this dataset, genes located on the HER2 amplicon show differential expression owing to the HER2 amplicon. As outlined in the Methods section, we designated 15 genes as positives, based on their expression values (i.e. over-expressed and co-expressed with HER2) and whether they reside on the HER2 amplicon on Chromosome 17q. Here we also used the PPV as the performance measure, i.e. scoring, for each method, the number of positives in the top *N* candidate list. The results for a range of top *N* values are depicted in Figure 4. From this figure, it is clear that DIDS outperforms all other methods over a wide range of values of *N*. While DIDS reported a median of nine positives in the top 20 (of an estimated 15, also see Supplementary Figure S1), the OS, ORT, COPA and the Mann–Whitney test reported a median of 0 of the genes from the amplicon. SAM, MOST and the *t*-test performed slightly better and reported median scores of 1 or 2. Of the different DIDS scoring functions, the 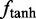 scoring function performed the best, with a mean performance of 9.15 of 15 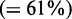.

### Results on the mouse dataset

**Figure 5.**
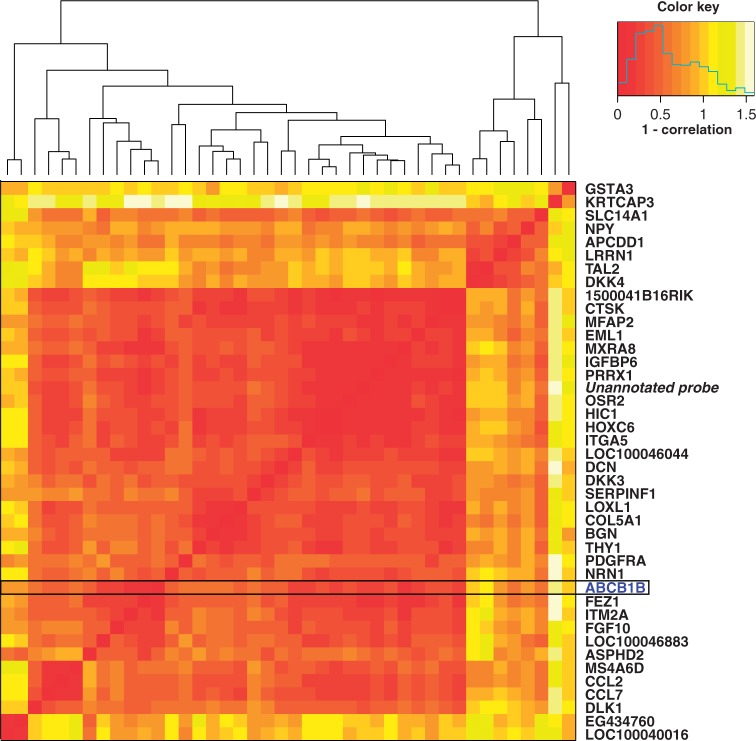
PPV comparison on the mouse dataset. Heatmap depicting the pairwise dissimilarity (1-Pearson correlation) between the top 50 docetaxel resistance markers detected by DIDS using the 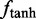 scoring function on the mouse dataset. DIDS ranked the gene ABCB1B (indicated by the black box) at position 30. ABCB1B has been confirmed to confer resistance to docetaxel in a subgroup of the resistant mouse tumors in a separate study. It is also clear from this dissimilarity matrix that within these top 50 candidates, 29 genes (36 probes) show very high similarity as represented by the prominent red block.

The third control set is derived from tumors that arose spontaneously in a mouse model that was genetically engineered to develop breast tumors. For a cohort of these mice, gene expression profiling was performed on primary tumors that were resistant as well as primary tumors that were sensitive to treatment with docetaxel. For this mouse model, it has been established that ABCB1B causes resistance to docetaxel treatment (6). We applied all approaches to the gene expression data derived from the primary tumors to retrieve putative resistance markers. Although DIDS ranks ABCB1B at position 30, the *t*-test, SAM and the other approaches ranked it much lower. The best performer of the other algorithms was the COPA algorithm, placing the ABCB1B gene at rank 255, whereas the others placed it at an even lower rank (mean position 2438, median position 2301). Furthermore, in a heatmap showing a clustering of the top 50 ranked genes (using Pearson correlation as the distance measure), it immediately becomes clear that most genes in the top 50 co-cluster with ABCB1B, indicating that these genes are part of a co-regulated gene cluster (Figure 5). Although no pathway including these genes was significantly enriched in the DAVID pathway analysis we performed (13), further research will focus on investigating common targets and drivers within this candidate list.

### Speed benchmark

To assess real world performance, we measured the time required by each approach to process the same dataset (25 000 genes, 100 versus 100 samples, 10 analyses). Table 1 shows that DIDS is faster than any other method, ranging from a factor 3.3 (compared with the *t*-test) to a factor 19.5 (compared with the MOST procedure). Also note that the MOST, OS, ORT and COPA procedures do not return a *P*-value and only give a single-sided result. In contrast, DIDS, SAM, the Mann–Whitney, KS test and the *t*-test output a two-sided result, i.e. reporting over- as well as under-expressed genes, and return an analytical exact *P*-value.

**Table 1.** Runtimes of benchmarked analytical approaches

Test	MOST	Mann–Whitney	OS	SAM	ORT	KS	COPA	*t*-test	DIDS
Time (s)	879	481	313	310	248	169	163	148	45
Factor difference	19.5	10.7	7.0	6.9	5.5	3.8	3.6	3.3	1

A total of 25 000 genes were analyzed 10 times with a total sample size of 200 (100 controls, 100 cases). The reported running times are in seconds and for all 10 runs combined. The analyses were performed using a 2.4-Ghz AMD Opteron processor.

## DISCUSSION

Traditional methods, like the *t*-test or SAM, are not well suited to detect features that show aberrant signal in only a subgroup of one of the two groups in the comparison (i.e. imbalanced differential signal). One context in which this scenario is relevant is the detection of markers of chemotherapy resistance. The identification of such markers has been a major goal in cancer research, but, unfortunately, little success has been achieved so far. Part of the reason could be that if only a fraction of all resistant samples share a particular common resistance mechanism, then conventional data analysis will not detect such a mechanism, regardless of the amount of data that are included. Another classical statistical test, the KS test, does detect differences other than differences in the mean, but the disadvantage of this method is that it detects any difference in distribution (e.g. also a difference in variance), whereas we are specifically looking for changes in a specific direction. Clearly, a different way of analyzing the data is required. The algorithm we propose stems from the intuitive notion that if a gene is involved in causing resistance to the treatment, then, almost by definition, the expression needs to be higher (or lower) in some non-responder samples compared with any of the responder group samples, i.e. if a responder would also show high (or low) expression of a resistance-causing gene, it would not have become a complete responder. The highest (or lowest) gene expression over all samples in the responder group then becomes the threshold from which the score is calculated in the non-responder group. Although the inspiration of the algorithm was drawn from the chemotherapy resistance context, it can be applied in any other situation in which only a subgroup of samples in the case group shows aberrant signal compared with the samples in the control group.

A number of methods were developed by others to tackle similar kinds of problems. An important difference between DIDS and these methods is the fact that DIDS is the only method to require all outlier samples to have expression higher (lower) than the maximum (minimum) in the control group. By setting this stringent threshold, we are assured that the resulting marker candidates offer a clear separation between cases and controls. In the context of chemotherapy resistance for example, aberrant expression of a marker gene cannot be equal to the expression of samples in the responder group. If that were the case, and the gene truly is a marker for resistance, that sample could not have been a responder. While some methods do base the threshold on samples in the control group only (ORT and MOST), other methods do not exploit this information at all (OS and COPA). In addition, DIDS can integrate different scoring functions leading to the detection of specific aberrant expression patterns. Finally, DIDS is the only subgroup-based method to provide an exact *P*-value. Conceptually and algorithmically, DIDS is also the most simple and intuitive method.

We showed that DIDS has higher power than all other methods, with the exception of cases with a high percentage of aberrant samples and for small difference between the mean aberrant and normal expression values. In these cases, the *t*-test and similar tests such as the Mann–Whitney test and SAM performed slightly better. In the scenario with 25 controls and 95 cases, DIDS tanh was outperformed in a single case: for 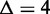 and 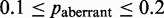 by COPA and OS in the power comparisons (Supplementary Figure S5). As a different scoring function can be plugged into DIDS to search for specific patterns (like a large expression difference in a small subgroup), we showed that the DIDS version using the quadratic scoring function did outperform COPA and OS (Supplementary Figure S6), even in this very specific scenario. However, the tanh-based scoring function was the most robust across all scenarios and types of tests, and we would recommend the tanh scoring function as the default scoring function. Interestingly, even for a scenario with a low number of samples (10 controls and 10 cases), DIDS managed to outperform all other methods, although this was only clearly the case for scenarios with relatively high signal-to-noise and a high percentage of aberrant cases (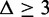 and 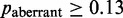; also see Supplementary Figure S4). The latter can be explained by the fact that in scenarios with small sample sizes, a low percentage of aberrant samples will translate to a low absolute number of aberrant cases (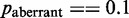 will result in only one aberrant case). Consequently, this will lead to lower power to detect these markers. To assess the influence of the different parameters (test used, Δ, 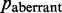, and α) on the power, we performed a three-way analysis of variance (ANOVA). All parameters and their interactions were highly significantly associated with power. The Δ, 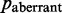, and the interaction of these two parameters with the test showed a particularly large influence on the power (see Supplementary Methods and Supplementary Tables S1–S4 for more details). Although the three-way ANOVA revealed that some factors (Δ, 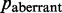, and their interaction) are more important than others to predict the power, it also showed that the relation between power and α, Δ, and 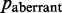 is complex (interactions between three factors are significant). Therefore, we believe that it is difficult to provide simple and general guidelines for practitioners based on the ANOVA model.

Next, we chose to focus on the PPV as a performance measure. In ranked-list candidate gene approaches, this is the most important and relevant performance measure because a high PPV will ensure that a high percentage of the identified candidates are true positives, which will lead to a higher success rate when candidate genes are validated downstream. Note that False-Discovery Rate (FDR), a measure often used in method comparisons, and PPV are closely related, specifically: FDR = 1 - PPV. Using two artificial datasets and an *in vivo* mouse dataset, we showed that DIDS outperformed all tested methods in terms of the PPV. Only for the scenario with 25 controls and 95 cases and 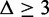 and 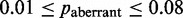 and the scenario with 10 controls and 10 cases for 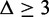 was DIDS tanh outperformed by COPA and OS (Supplementary Figures S9 and S11). When we used DIDS with the quadratic scoring function in the 10 controls and 10 cases scenario, DIDS was superior to all other methods (Supplementary Figure S10). Especially noteworthy is that in the real-world mouse model dataset the other algorithms managed to rank the previously validated resistance marker, ABCB1B, only at position 255, at best, whereas DIDS placed this gene at position 30. While we have focused on DIDS as a strategy for identifying single gene biomarkers, additional pathway analysis can be performed to assess whether the identified candidates interact through specific biological pathways. Unfortunately, for the mouse dataset in our study, pathway analysis of the top 50 candidate genes did not lead to any additional insights.

The context of analyzing gene expression data to predict resistance to chemotherapy in cancer clearly showcases the capabilities of DIDS. However, because it is a general methodology, the approach can be applied in many different contexts. One immediate and obvious extension would be to apply DIDS to other genomics data, such as aCGH or protein expression, to identify resistance mechanisms or to identify subtypes of cancer. In a broader sense, another field of application could be antibiotics resistance. Resistance to antibiotics in bacterial species is a typical situation where distinct populations will show different mechanisms of resistance (14). If a set of bacterial colonies would be analyzed to identify resistance mechanisms, traditional analytical methods might fail for the exact same reasons as it would in the case of chemotherapy resistance. An approach such as DIDS, that takes heterogeneity in the marker signal into account, would be preferred.

## CONCLUSION

Because traditional methods are far from optimal to detect features that show differential signal, compared with a control group, in only a subgroup of the case group, we have developed an algorithm that is much better at detecting such features. We showed that this is relevant by giving a real world example of detecting markers of chemotherapy resistance. We showed in simulation studies that, for the conditions outlined here, DIDS outperforms all other methods, including methods also designed to deal with these types of data, in terms of both power and PPV. For case versus control comparisons in which the case group is heterogeneous, out of all included approaches DIDS is the best performing method.

## SUPPLEMENTARY DATA

Supplementary Data are available at NAR Online.

## FUNDING

Funding for open access charge: CTMM, the Center for Translational Molecular Medicine (www.ctmm.nl), project Breast CARE [03O-104].

*Conflict of interest statement*. None declared.

## Supplementary Material

Supplementary Data

## References

[1] Borst P, Wessels L (2010). Do predictive signatures really predict response to cancer chemotherapy?. Cell Cycle.

[2] Popovici V, Chen W, Gallas BG, Hatzis C, Shi W, Samuelson FW, Nikolsky Y, Tsyganova M, Ishkin A, Nikolskaya T (2010). Effect of training-sample size and classification difficulty on the accuracy of genomic predictors. Breast Cancer Res..

[3] Tusher VG, Tibshirani R, Chu G (2001). Significance analysis of microarrays applied to the ionizing radiation response. Proc. Natl Acad. Sci. USA.

[4] de Ronde JJ, Hannemann J, Halfwerk H, Mulder L, Straver ME, Vrancken Peeters MJ, Wesseling J, van de Vijver M, Wessels LF, Rodenhuis S (2010). Concordance of clinical and molecular breast cancer subtyping in the context of preoperative chemotherapy response. Breast Cancer Res. Treat.

[5] Rodrigues AS, Dinis J, Gromicho M, Martins C, Laires A, Rueff J (2012). Genomics and cancer drug resistance. Curr. Pharm. Biotechnol..

[6] Rottenberg S, Vollebergh MA, de Hoon B, de Ronde J, Schouten PC, Kersbergen A, Zander SA, Pajic M, Jaspers JE, Jonkers M (2012). Impact of intertumoral heterogeneity on predicting chemotherapy response of BRCA1-deficient mammary tumors. Cancer Res..

[7] MacDonald JW, Ghosh D (2006). COPA–cancer outlier profile analysis. Bioinformatics.

[8] Tomlins SA, Rhodes DR, Perner S, Dhanasekaran SM, Mehra R, Sun XW, Varambally S, Cao X, Tchinda J, Kuefer R (2005). Recurrent fusion of TMPRSS2 and ETS transcription factor genes in prostate cancer. Science.

[9] Tibshirani R, Hastie T (2007). Outlier sums for differential gene expression analysis. Biostatistics.

[10] Wu B (2007). Cancer outlier differential gene expression detection. Biostatistics.

[11] Lian H (2008). MOST: detecting cancer differential gene expression. Biostatistics.

[12] Dekker TJ, Borg ST, Hooijer GK, Meijer SL, Wesseling J, Boers JE, Schuuring E, Bart J, van Gorp J, Mesker WE (2012). Determining sensitivity and specificity of HER2 testing in breast cancer using a tissue micro-array approach. Breast Cancer Res. Treat.

[13] Huang DW, Sherman BT, Lempicki RA (2009). Bioinformatics enrichment tools: paths toward the comprehensive functional analysis of large gene lists. Nucleic Acids Res..

[14] Andersson DI, Hughes D (2012). Evolution of antibiotic resistance at non-lethal drug concentrations. Drug Resist. Updat..

