# Supplementary material for: Identifying subgroup markers in heterogeneous populations
Source: Nucleic Acids Res. 2013 Sep 20;41(21):e200. doi: 10.1093/nar/gkt845 (PMC3834840; doi:10.1093/nar/gkt845)
Supplement: Supplementary Data [file supp_41_21_e200__index.html]

Identifying subgroup markers in heterogeneous populations — Identifying subgroup markers in heterogeneous populations — Supplementary Data 

# Identifying subgroup markers in heterogeneous populations

## Supplementary Data

files

**Files in this Data Supplement:**

- Supplementary Data - pdf file
