# Supplementary material for: Identifying subgroup markers in heterogeneous populations
Source: Nucleic Acids Res. 2013 Sep 20;41(21):e200. doi: 10.1093/nar/gkt845 (PMC3834840; doi:10.1093/nar/gkt845)
Supplement: Supplementary Data [file supp_gkt845_nar-01329-met-n-2013-File001.pdf]

Supplemental Material

Supplemental Methods

DIDS algorithm for imbalanced differential underexpression

Supplemental Equation 1 shows the DIDS score for genes that are differentially underexpressed compared to the control group.

$$j_{\text{down}} = \sum_{i=1}^{n_2} f(|\check{x}_1 - x_{2i}|^+) \tag{1}$$

with

$$\check{x}_1 = \min_i \{x_{1i}\}$$

DIDS permutation based P-value calculation

In this section we show that it is possible to compute a permutation based p-value for the DIDS statistics without explicitly considering all possible relabelings of the samples. More specifically, we derive an explicit closed-form formula for this p-value.
For a particular sample we want to assess whether the observed DIDS score, j_{up}, as defined in Equation 1 is unexpectedly high or not. More precisely, we want to compute, under H_0, the probability $P(J_{\text{up}} \geq j_{\text{up}})$, where j_{up} is the observed value of J_{up}.

Resampling strategy. Under H_0 the two groups are drawn from the same distribution. In other words, we construct a new data set by drawing, at random and without replacement, n_1 samples from the complete set of $n = n_1 + n_2$ samples. We assign these n_1 samples to the control group. The n_2 remaining samples are assigned to the case group.

Possible values and p-values of the score For the sake of simplicity we consider that the $n = n_1 + n_2$ observed samples are ordered and we denote them: $y_1 \geq y_2 \ldots \geq y_n$. We also assume that all these values are different such that we have: $y_1 > y_2 \ldots > y_n$. For one particular data set we see that if we know that y_k is the observed value of $\hat{X}_1 : \hat{x}_1$ then there is only one possible value for the score. Indeed, all samples in the case group that are smaller than y_k do not contribute to the score. Furthermore, it follows that all $\{y_i\}_{i<k}$ belong to the case group. If that were not the case, the maximum of the control group would not be y_k. From this we get that the only possible score if $y_k = \hat{x}_1$ is

$$j_{\text{up}} = \sum_{i=1}^{k} f(|y_i - y_k|^+).$$

We call this value $j_{\text{up}}(y_k)$. From this we see that the number of possible values for the score J_{up} is smaller or equal to the number of possible values for $\hat{X}_1$. $\hat{X}_1$ can only assume the following value: $\{y_i\}_{i \leq n_2+1}$ (that is, if all $X_{1i} < X_{2i}$ then $y_k = y_{n2+1}$ and if all $X_{1i} > X_{2i}$, then $y_k = y_1$).

As y_i are different from each other and assuming $f(x) > 0$ for $x > 0$ we get that J_{up} can take $n_2 + 1$ possible values. For any $i \leq n_2 + 1$ we get by straightforward combinatorial arguments that under H_0 :

$$P_{H_0}(\hat{X}_1 = y_i) = \binom{n_1 + n_2 - i}{n_1 - 1} / \binom{n_1 + n_2}{n_1}.$$

We can now compute a p-value for the score as follows:

$$P_{H_0}(J_{\text{up}} \geq j_{\text{up}}(y_i)) = \sum_{k=i}^{n_2+1} P_{H_0}(\hat{X} = y_k).$$

Importantly, the only thing that counts to calculate this p-value is the number of samples in the case group that are above the maximum of the control group, not the actual signals in the case group. From this it is clear that the ordering of the genes obtained using the p-values can be quite different from the ordering obtained using the actual DIDS score. Furthermore, given that this permutation based p-value only depends on the number of samples in the case group above the maximum of the control group, it is quite clearly underpowered. However we do not use this p-value calculation as a way to control the Type I error rate, but rather as a way to discard genes that are supported by very few samples as explained in the next sub-section.

Heuristic filtering of results

We use the analytically computed exact p-value (as outlined in the previous section) to remove genes that have a high DIDS score but a low number of supporting samples, i.e. genes with high excess expression in a small number of cases. Since we know this p-value is a very conservative estimate of the true p-value and we employ the p-value as a heuristic filter (we finally rank by the DIDS score, not the p-value), we do not apply multiple testing correction. Therefore, the p-values obtained by DIDS should not be directly compared to p-values derived with other methods, but should purely be used to filter the results. We set the default p-value threshold to α = 0.05, and remove all genes with a p-value exceeding this threshold. The positive effect on accuracy is most notable for scoring functions that are biased towards large excess expression in relatively few samples (for example the quadratic scoring function). Supplemental Figure 3 illustrates this effect clearly. This figure shows the PPV as a function of the top N candidate reporters, for the case where no p-value filtering is applied. From this figure it is clear that the tanh and sqrt PPV curves are nearly identical to their filtered counterparts as depicted in Figure 4. In contrast, the PPV curve for the quad scoring function shows a clear drop in performance when no filtering is applied.

Relation between power and PPV

The link between power and PPV is important, but non-trivial. The statistical power can we written as the expected value of the ratio of the number of true positives (TP) and the sum of the number of true positives and false positives (FP), given a confidence level, α. More formally, the power is given by: $E(TP/(TP + FP)|\alpha))$. On the other hand, the PPV is the same ratio, but given the number of selected top genes, N. Specifically, the PPV is given by

$E(TP/(TP+FP)|N)$). From this it is clear that PPV and power are related. If we have a good estimation of alpha (which is not necessarily the case, as this depends on an accurate definition of the null distribution) the power and PPV analysis should provide very similar results. For screening purposes, however, the PPV makes more intuitive sense. For this reason we decided to focus on the PPV as performance measure.

Three-way ANOVA power analysis

We performed a three-way ANOVA in order to assess the importance of the different factors on the power. For each simulated scenario (10 controls versus 10 cases, 25 versus 95, 50 versus 100, and 100 versus 100) we assessed the power of each test that we used for different α thresholds ($0.05 * (1/2)^k$ for k in 1 to 11). In this way we obtained four tables (one for each scenario) containing the power as a function of the test used to perform the analysis, the α threshold, the difference between aberrant cases and controls (Δ) and the percentage of aberrant cases (p_{aberrant}). This resulted in a full factorial design (i.e. we have a measurement of the power for every combination of test, α, Δ, and p_{aberrant}).
We considered all possible interactions between two and three factors. If we call $Y_{t,\alpha,\Delta,p}$ the power for the test t, our ANOVA model can be written as :

$$\begin{aligned} &Y_{t,\alpha,\Delta,p_{\text{aberrant}}} = \\ &\mu + a_t + b_\alpha + c_\Delta + d_{p_{\text{aberrant}}} + e_{t,\alpha} + f_{t,\Delta} + g_{t,p_{\text{aberrant}}} + h_{\alpha,\Delta} + i_{\alpha,p_{\text{aberrant}}} + j_{\Delta,p_{\text{aberrant}}} + \\ &\qquad k_{t,\alpha,\Delta} + l_{t,\alpha,p_{\text{aberrant}}} + m_{t,\Delta,p_{\text{aberrant}}} + n_{\alpha,\Delta,p_{\text{aberrant}}} + \varepsilon_{t,\alpha,\Delta,p_{\text{aberrant}}} \end{aligned} \quad (2)$$

We observed that all factors and their interactions were highly significant. Nonetheless, some of these factors and interactions had a, relatively, much larger sum of squares. In particular, except for the 10 versus 10 scenario where the power was low overall:
1.) Δ, p_{aberrant} and their interaction had a large sum of squares (>100)
2.) The α threshold on its own was also associated with a large sum of squares (>100). This would, in general, be expected.
3.) The interaction between the test (t) and Δ, as well as the interaction between t and p_{aberrant} were also associated with a large sum of squares ($\geq$ 100).
We conclude from (1) that the percentage of aberrant cases (p_{aberrant}) and the difference between controls and aberrant cases (Δ) have a large influence on the power and from (3) that the performances of the different tests clearly depend on p_{aberrant} and Δ. The sum of squares for all four scenarios can be found in Supplemental Tables 1, 2, 3, and 4.

Supplemental Figures

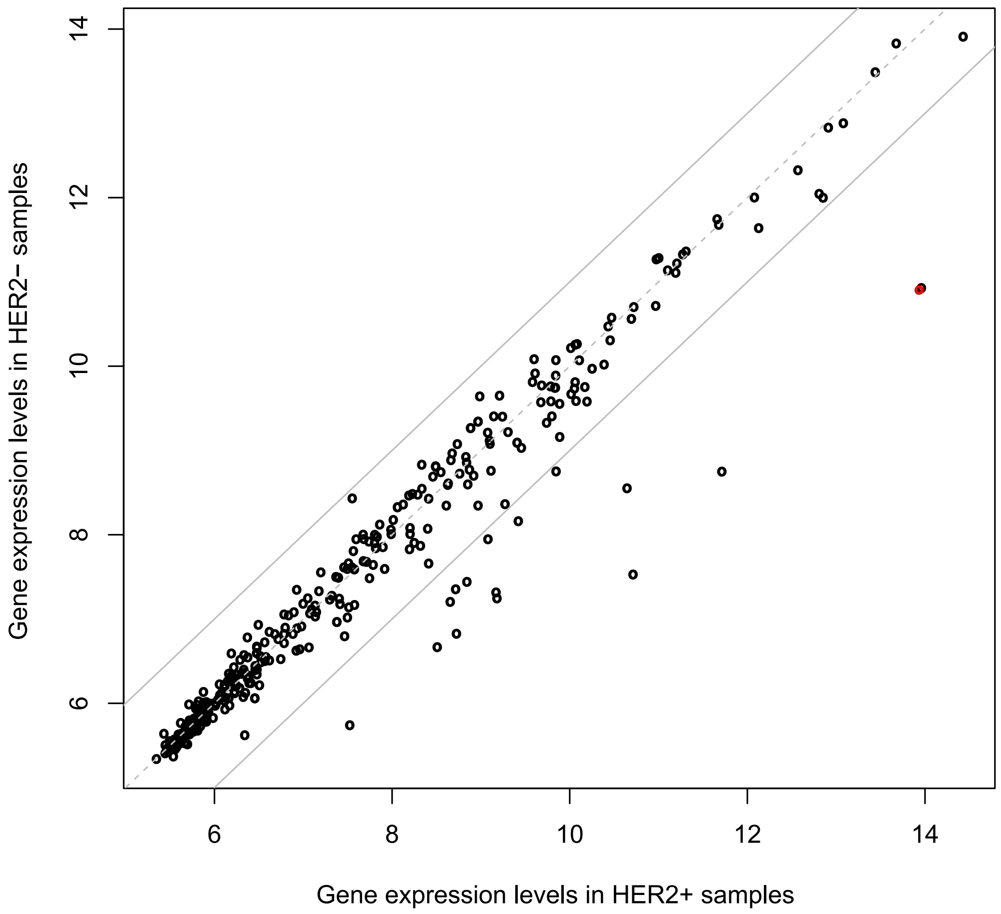

Supplemental Figure 1: mRNA expression of HER2-amplicon contained genes. Expression of genes on Chromosome 17q12 and 17q21. Of the 339 genes in these regions, 15 show a differential expression greater than one (log2 scale) when comparing HER2+ to HER2- samples. A log2 difference of one is indicated by the grey lines and the red circled dot represents the ERBB2 gene.

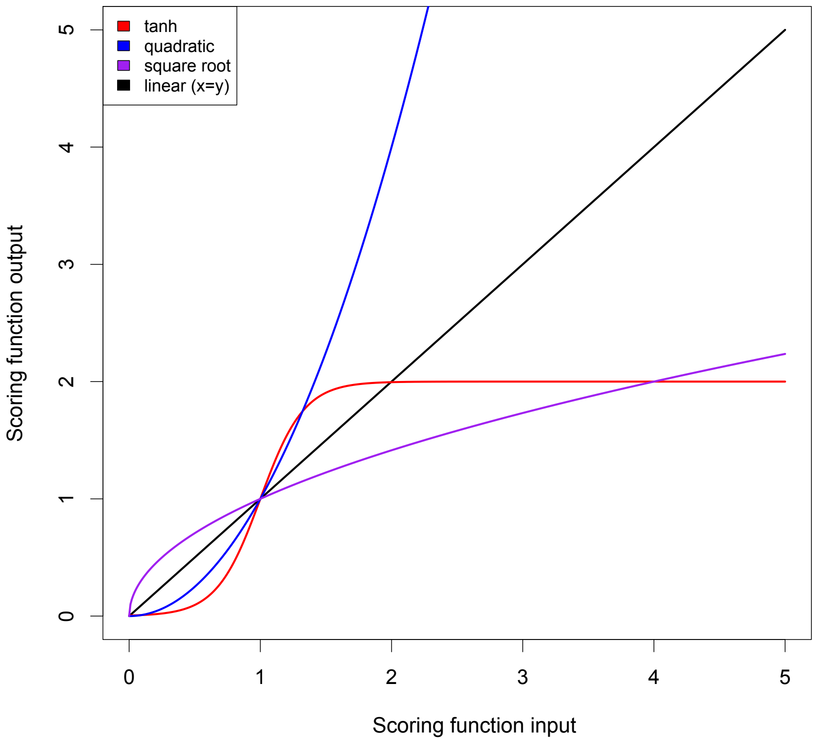

Supplemental Figure 2: Scoring functions implemented in DIDS. The three different scoring functions are suited for detecting different patterns of aberrant gene expression. The tangent based method ('tanh') weighs small excess expression values (< 1) slightly lower than a linear function, differences between one and two slightly more than the linear case and all differences larger than two are capped at two (note that the standard deviation over all genes in our breast cancer dataset is 1.85 and the inter-quartile range (IQR) is 2.88). It is our experience that this scoring function detects most aberrant patterns. The quadratic scoring function ('quadratic') penalizes small differences (< 1), but exaggerates differences larger than one. This scoring function is suited to detect patterns of infrequent aberrant samples that show a large difference compared to the control group. Finally, the square-root function ('square root') dampens the excess expression exceeding one compared to a linear scoring function and is suited to find patterns where a relatively large number of samples show an aberrant pattern in the case group.

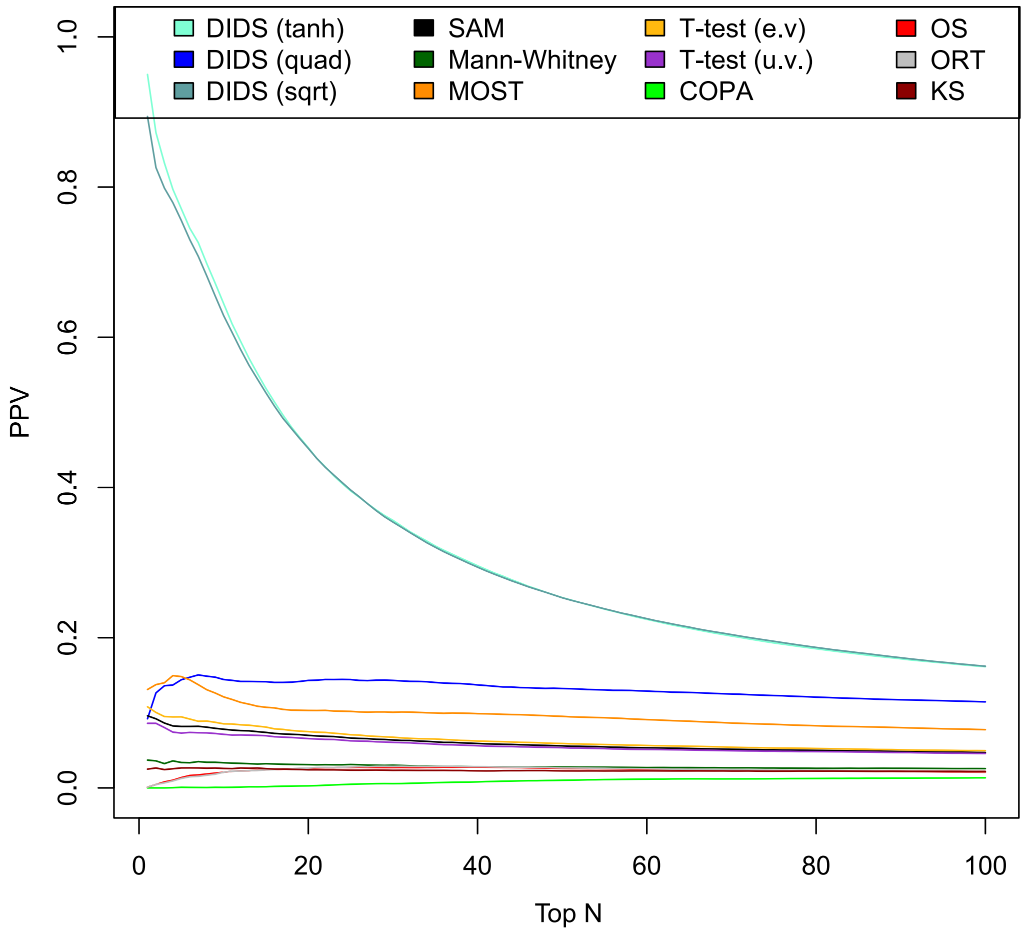

Supplemental Figure 3: PPV comparison on HER2 dataset for unfiltered DIDS scores. Positive predictive values (PPVs) for all algorithms on the HER2 dataset as a function of the top N candidates. The different variants of DIDS employing the different scoring functions are denoted by 'DIDS (tanh)', 'DIDS (quad)' and 'DIDS (sqrt)', respectively. This figure shows the performance when no p-value filtering is applied to the DIDS scores.

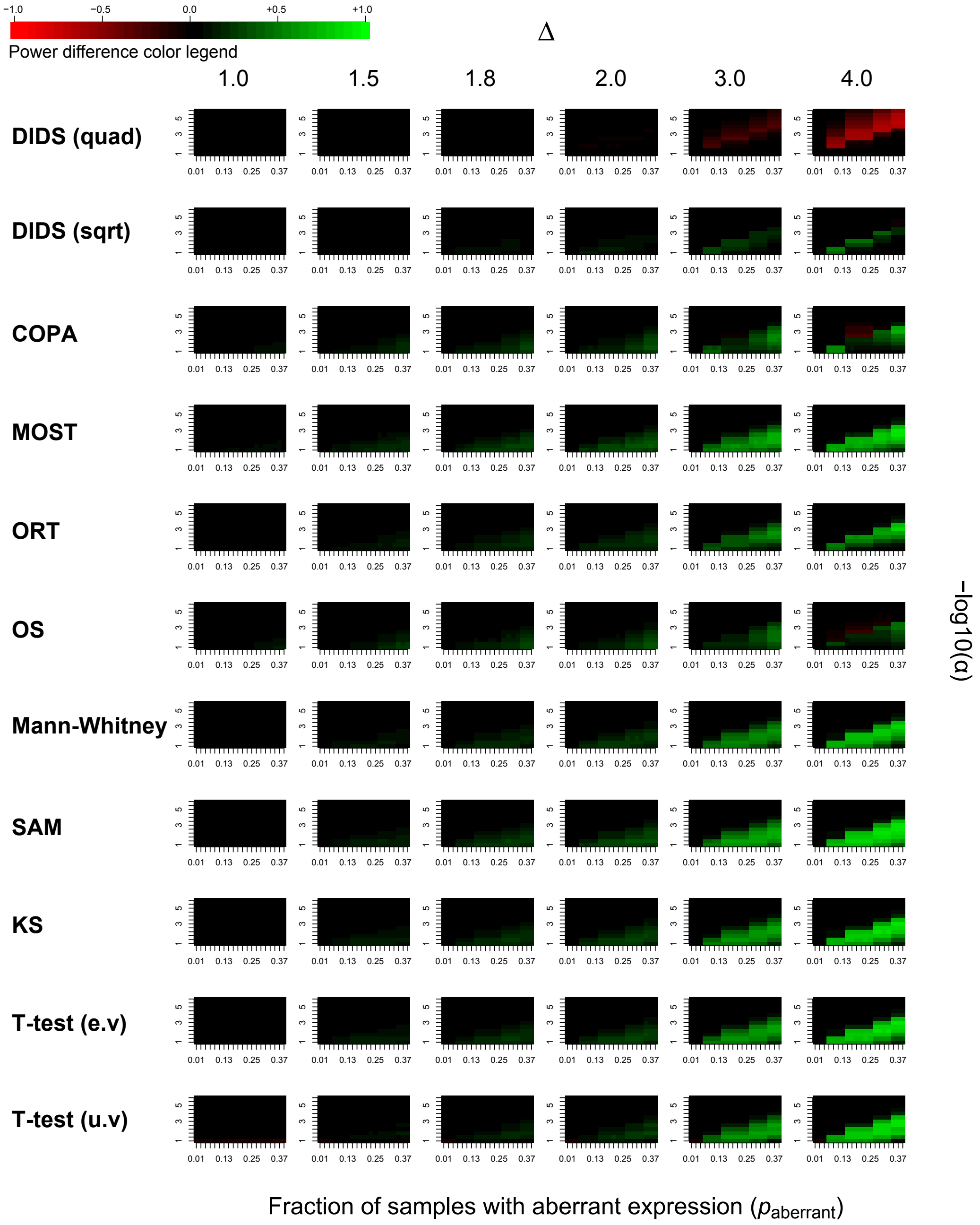

Supplemental Figure 4: The difference in power between DIDS and all other tested approaches for the scenario ($n_1 = 10; n_2 = 10$). The methods are represented in the rows and the different values of Δ in the columns. For each combination of a method and a value of Δ, the power difference for DIDS and the method represented in the column are depicted as a function of p_{aberrant} (horizontal axis) and α (vertical axis). Shades of green (red) represent settings where DIDS has more (less) power than the other methods.

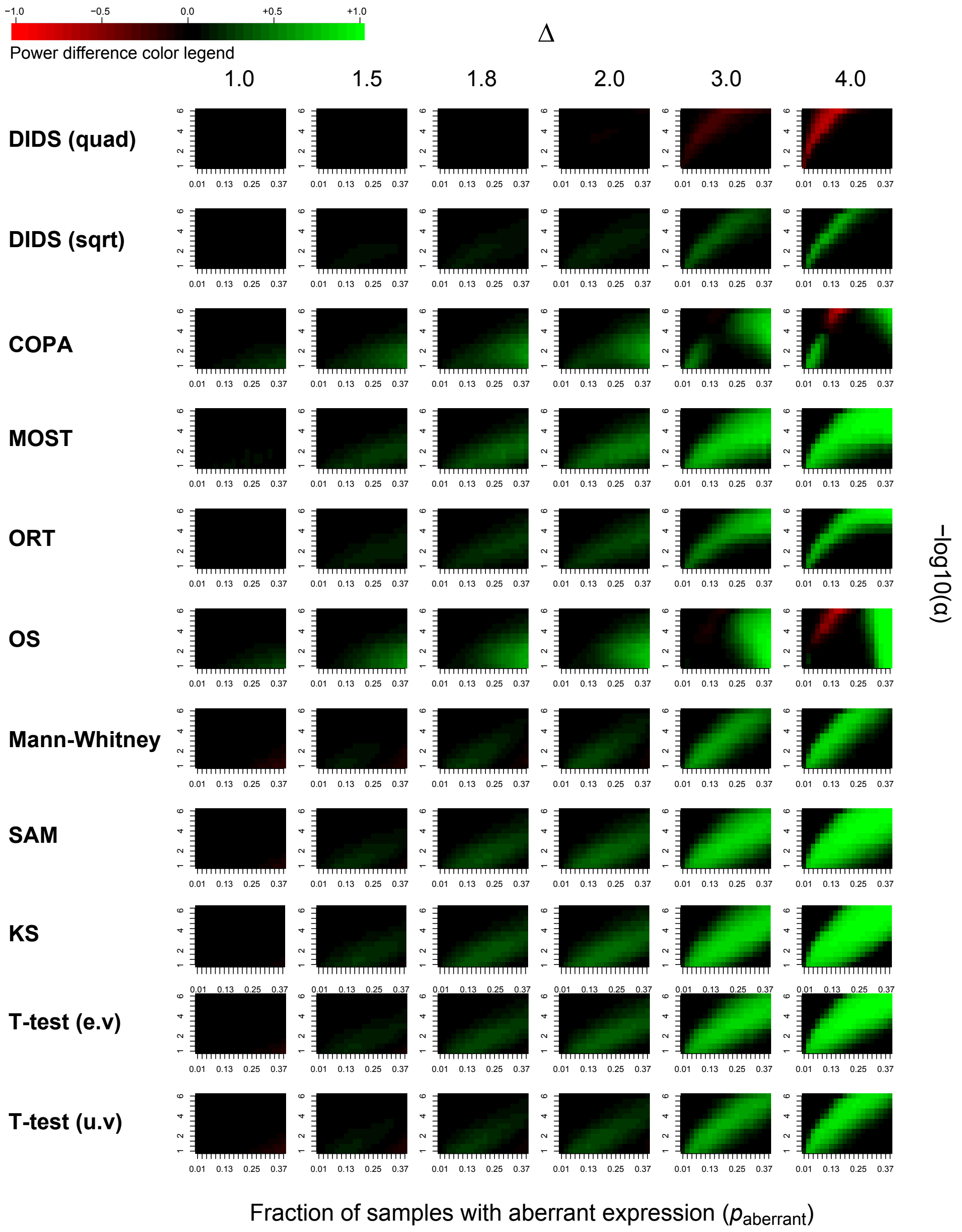

Supplemental Figure 5: The difference in power between DIDS and all other tested approaches for the scenario ($n_1 = 25; n_2 = 95$). The methods are represented in the rows and the different values of Δ in the columns. For each combination of a method and a value of Δ, the power difference for DIDS and the method represented in the column are depicted as a function of p_{aberrant} (horizontal axis) and α (vertical axis). Shades of green (red) represent settings where DIDS has more (less) power than the other methods.

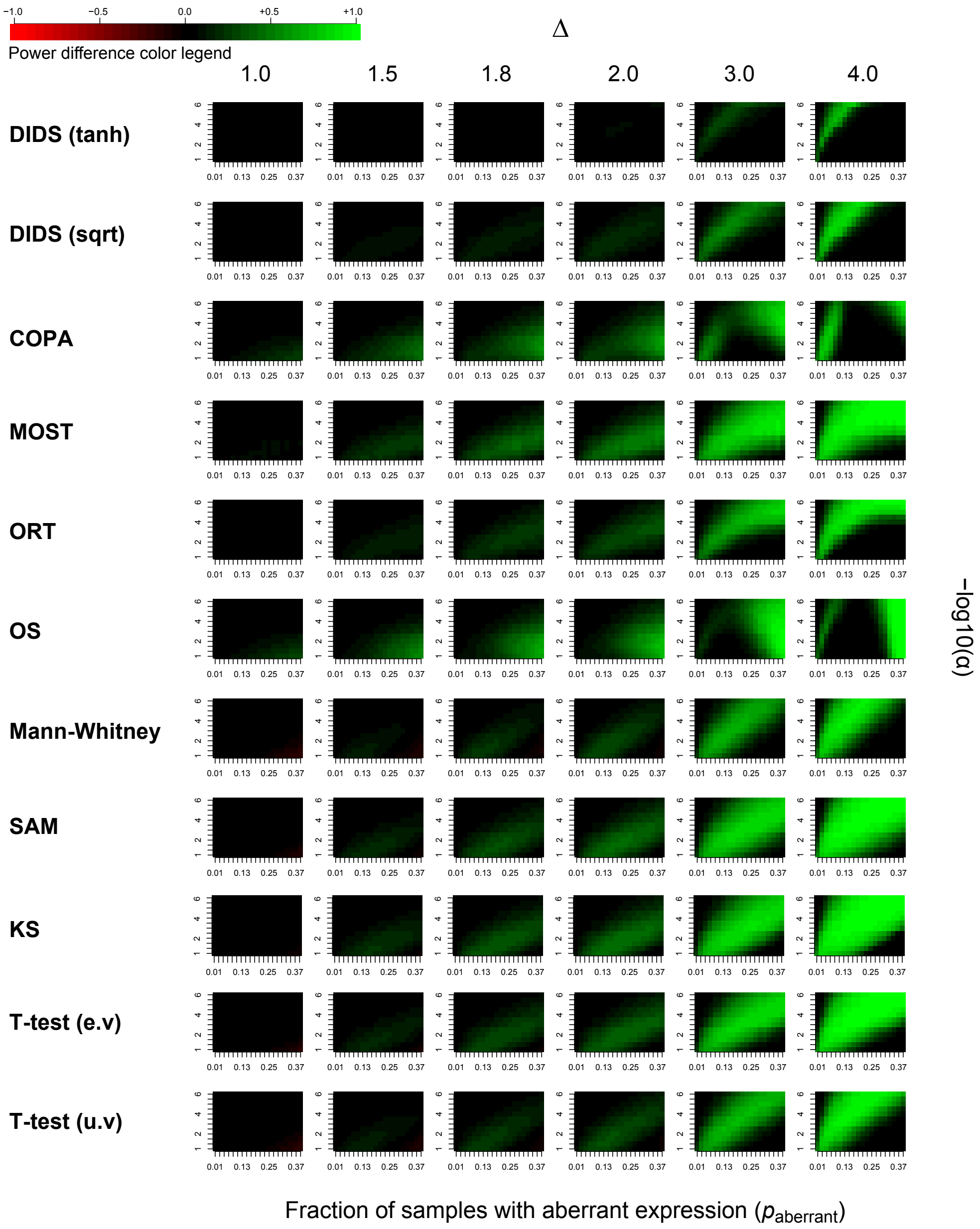

Supplemental Figure 6: The difference in power between DIDS and all other tested approaches for the scenario ($n_1 = 25; n_2 = 95$) **for the *quadratic* scoring function.** For this scenario, DIDS outperforms all other tests in all settings, including OS and COPA even for $\Delta = 4$. The methods are represented in the rows and the different values of Δ in the columns. For each combination of a method and a value of Δ, the power difference for DIDS and the method represented in the column are depicted as a function of p_{aberrant} (horizontal axis) and α (vertical axis). Shades of green (red) represent settings where DIDS has more (less) power than the other methods.

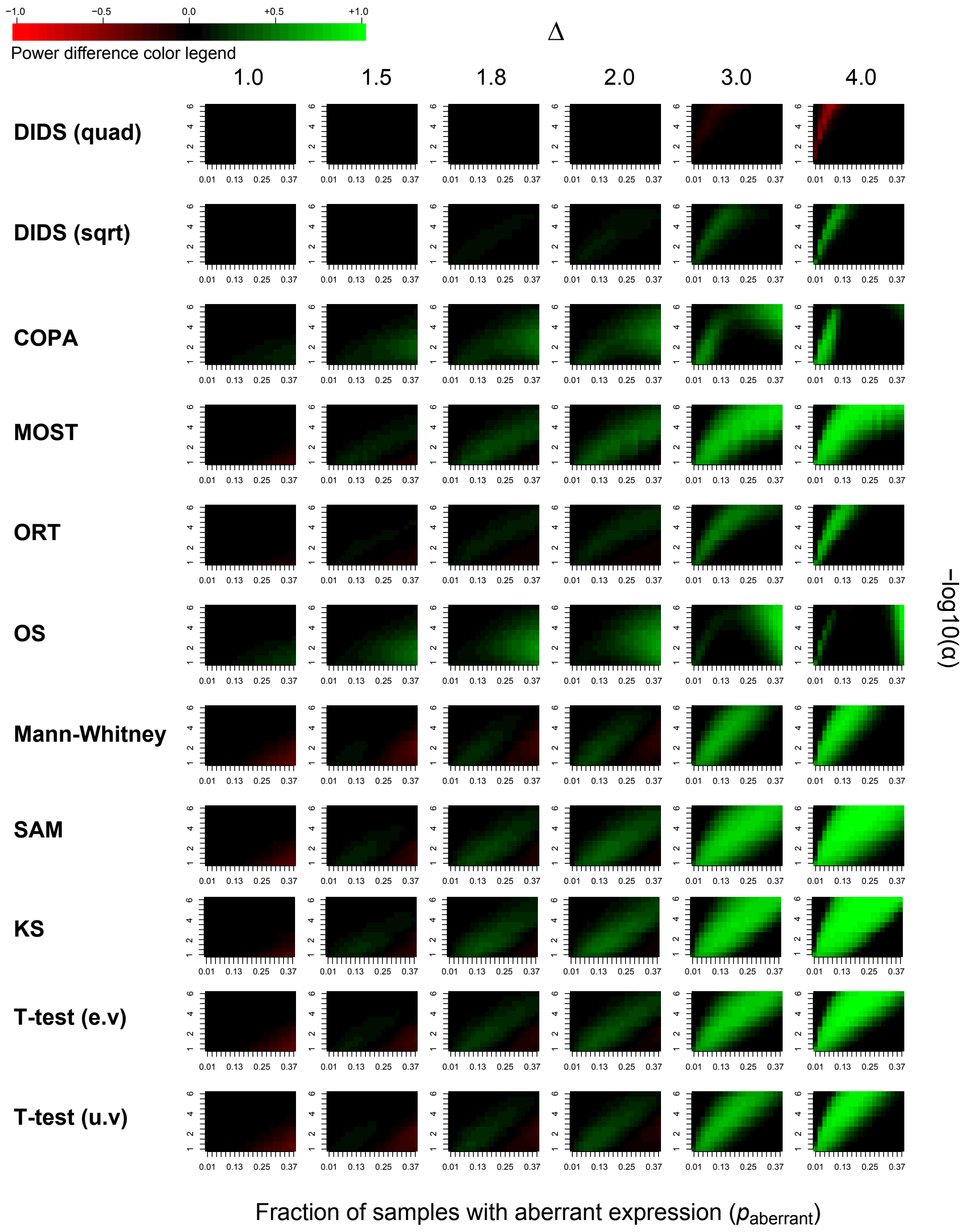

Supplemental Figure 7: The difference in power between DIDS and all other tested approaches for the scenario ($n_1 = 50; n_2 = 100$). The methods are represented in the rows and the different values of Δ in the columns. For each combination of a method and a value of Δ, the power difference for DIDS and the method represented in the column are depicted as a function of p_{aberrant} (horizontal axis) and α (vertical axis). Shades of green (red) represent settings where DIDS has more (less) power than the other methods.

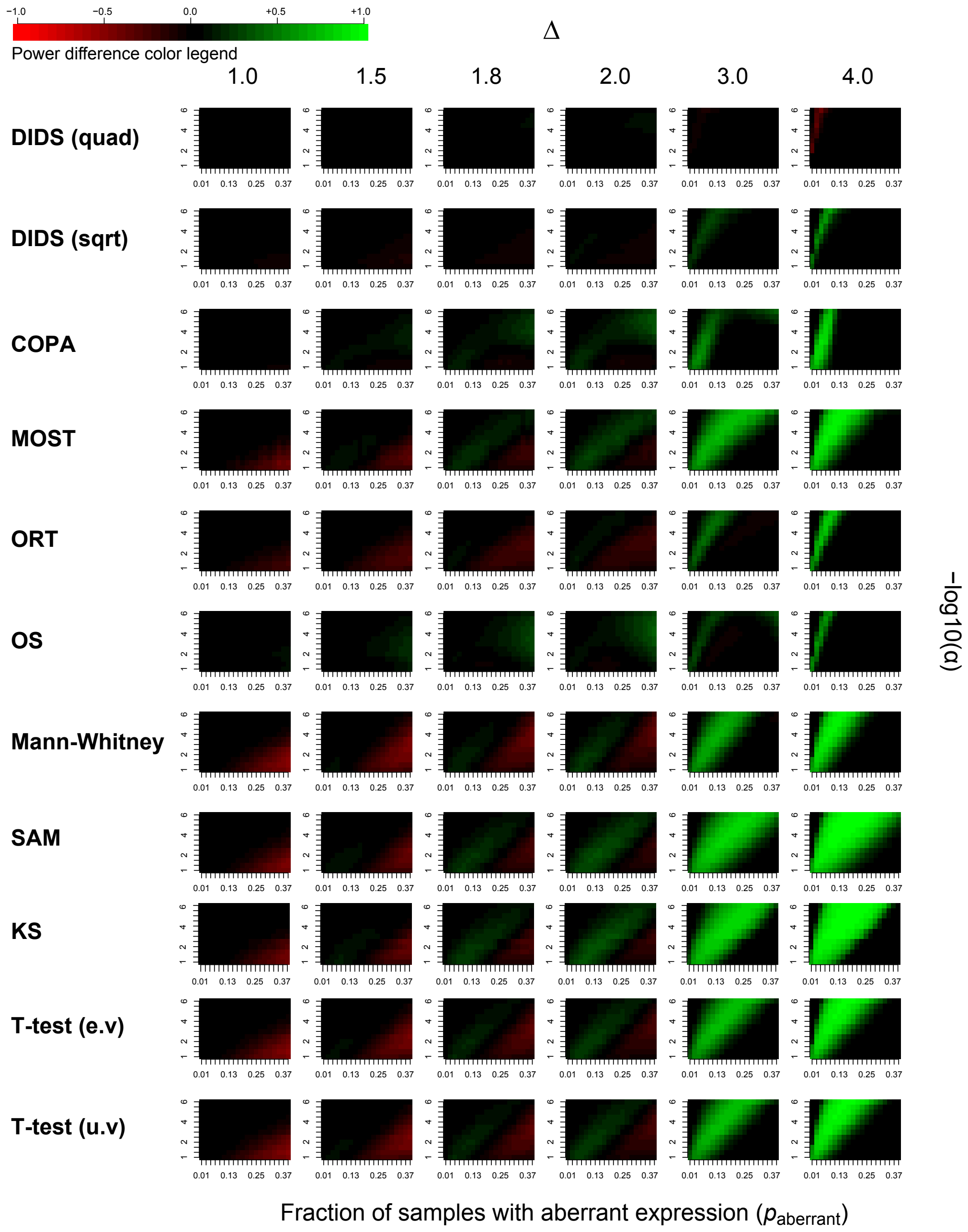

Supplemental Figure 8: The difference in power between DIDS and all other tested approaches for the scenario ($n_1 = 100; n_2 = 100$). The methods are represented in the rows and the different values of Δ in the columns. For each combination of a method and a value of Δ, the power difference for DIDS and the method represented in the column are depicted as a function of p_{aberrant} (horizontal axis) and α (vertical axis). Shades of green (red) represent settings where DIDS has more (less) power than the other methods.

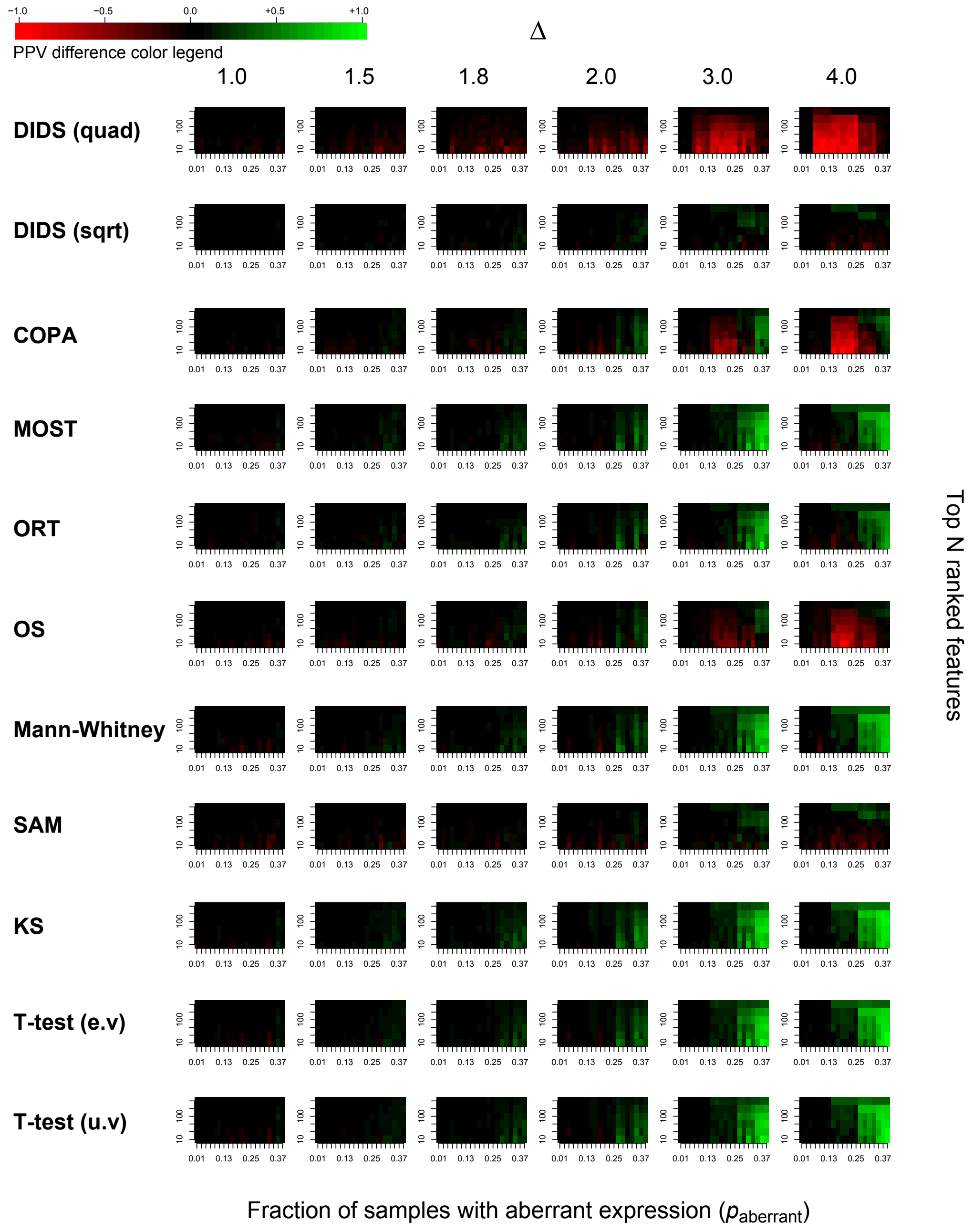

Supplemental Figure 9: The difference in PPV between DIDS and all other tested approaches for the scenario ($n_1 = 10; n_2 = 10$). The methods are represented in the rows and the different values of Δ in the columns. For each combination of a method and a value of Δ, the PPV differences for DIDS and the method represented in the column are depicted as a function of p_{aberrant} (horizontal axis) and α (vertical axis). Shades of green (red) represent settings where DIDS achieves a higher (lower) PPV than the other methods.

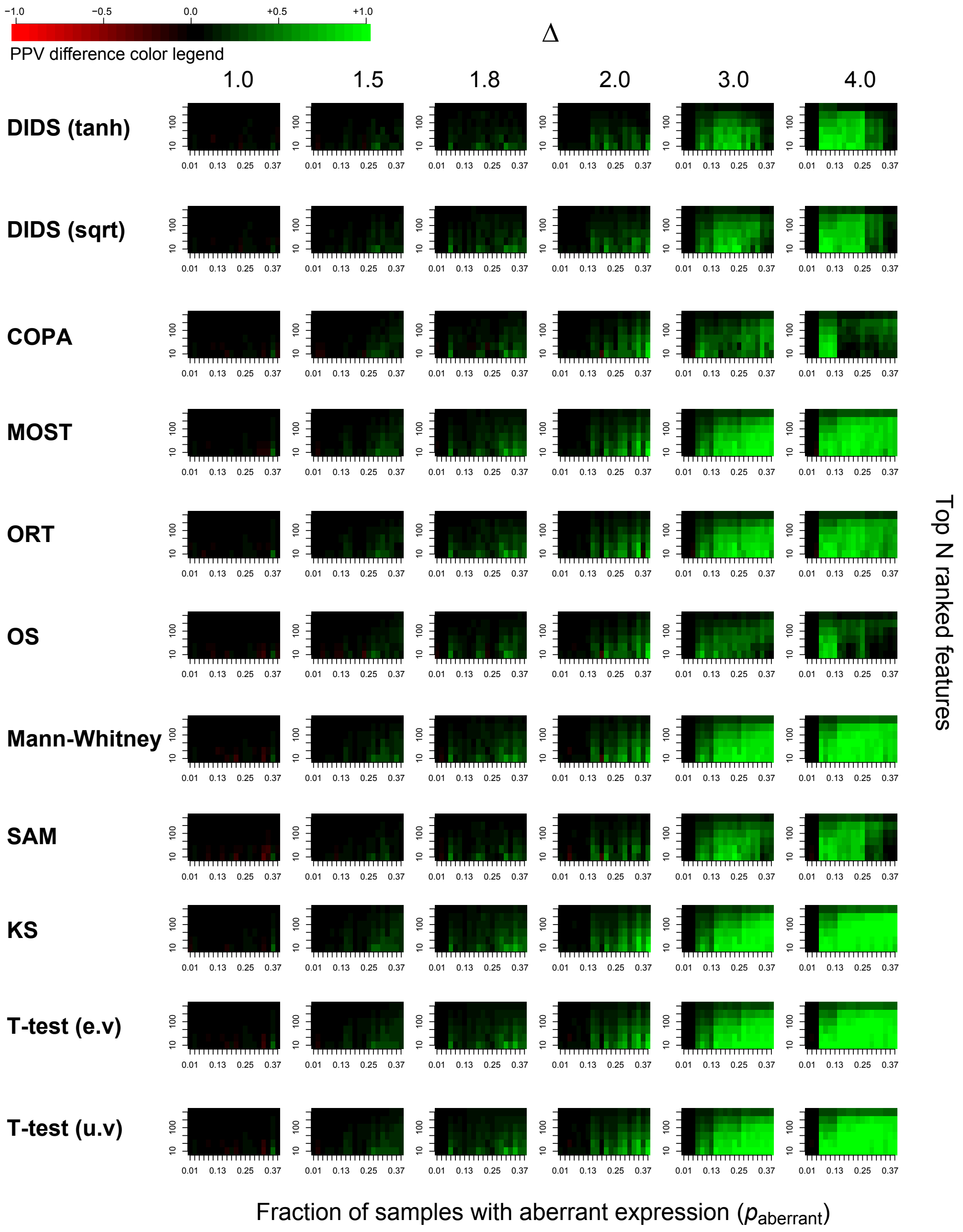

Supplemental Figure 10: The difference in PPV between DIDS and all other tested approaches for the scenario $(n_1 = 10; n_2 = 10)$ **for the *quadratic* scoring function.**. For this scenario, DIDS outperforms all other tests in all settings, including OS and COPA even for $\Delta \geq 3$. The methods are represented in the rows and the different values of Δ in the columns. For each combination of a method and a value of Δ, the PPV differences for DIDS and the method represented in the column are depicted as a function of p_{aberrant} (horizontal axis) and α (vertical axis). Shades of green (red) represent settings where DIDS achieves a higher (lower) PPV than the other methods.

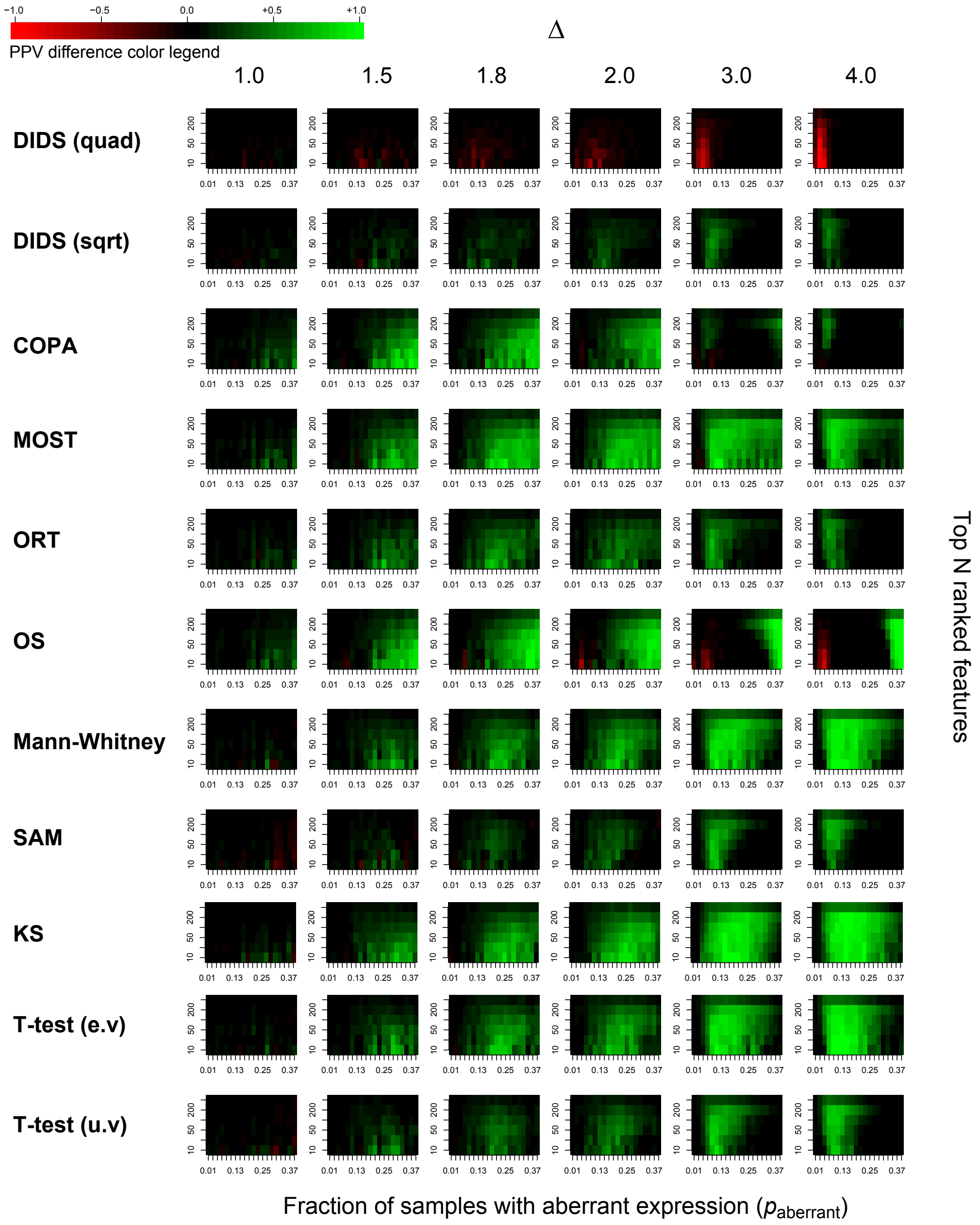

Supplemental Figure 11: The difference in PPV between DIDS and all other tested approaches for the scenario ($n_1 = 25; n_2 = 95$). The methods are represented in the rows and the different values of Δ in the columns. For each combination of a method and a value of Δ, the PPV differences for DIDS and the method represented in the column are depicted as a function of p_{aberrant} (horizontal axis) and α (vertical axis). Shades of green (red) represent settings where DIDS achieves a higher (lower) PPV than the other methods.

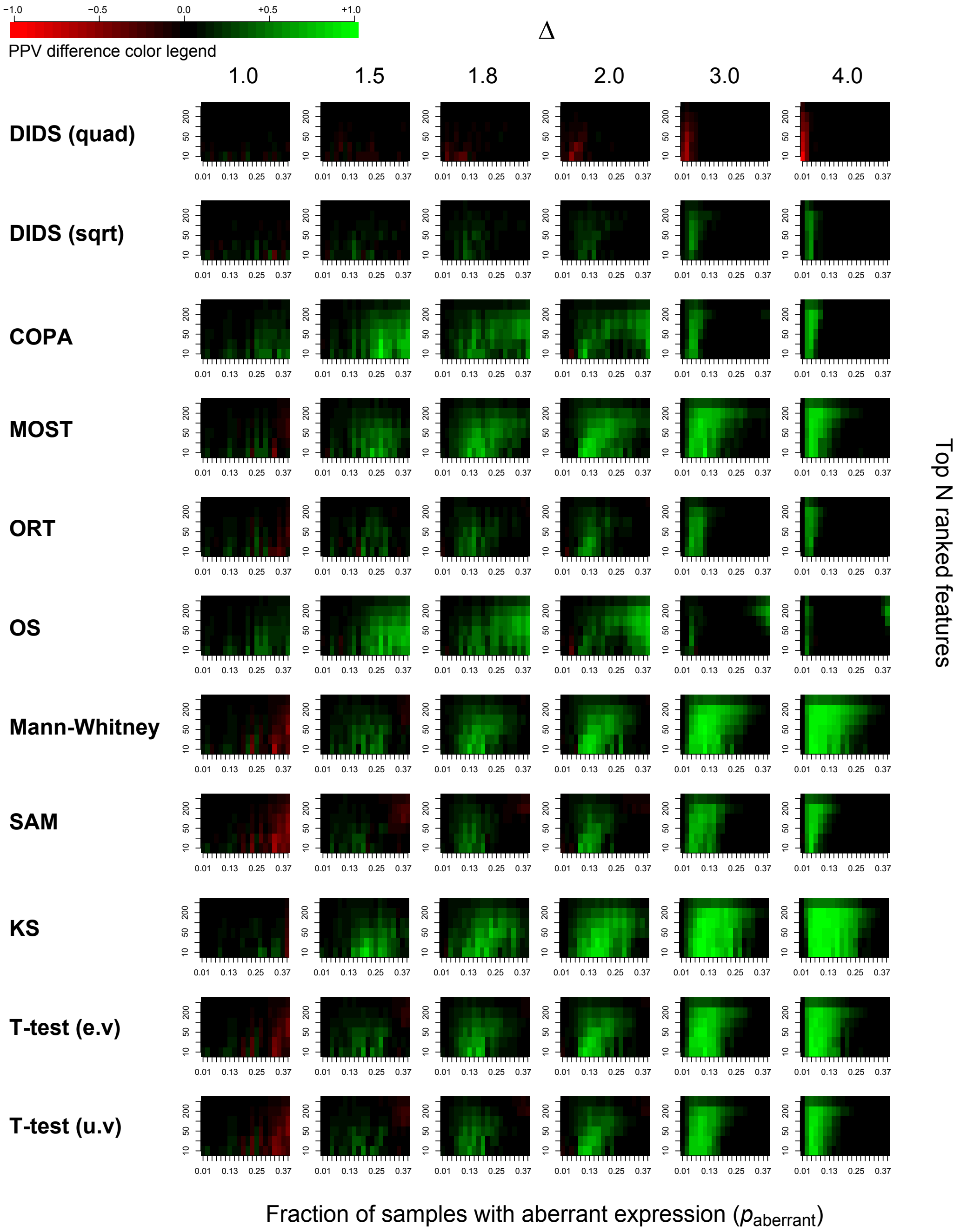

Supplemental Figure 12: The difference in PPV between DIDS and all other tested approaches for the scenario ($n_1 = 50; n_2 = 100$). The methods are represented in the rows and the different values of Δ in the columns. For each combination of a method and a value of Δ, the PPV differences for DIDS and the method represented in the column are depicted as a function of p_{aberrant} (horizontal axis) and α (vertical axis). Shades of green (red) represent settings where DIDS achieves a higher (lower) PPV than the other methods.

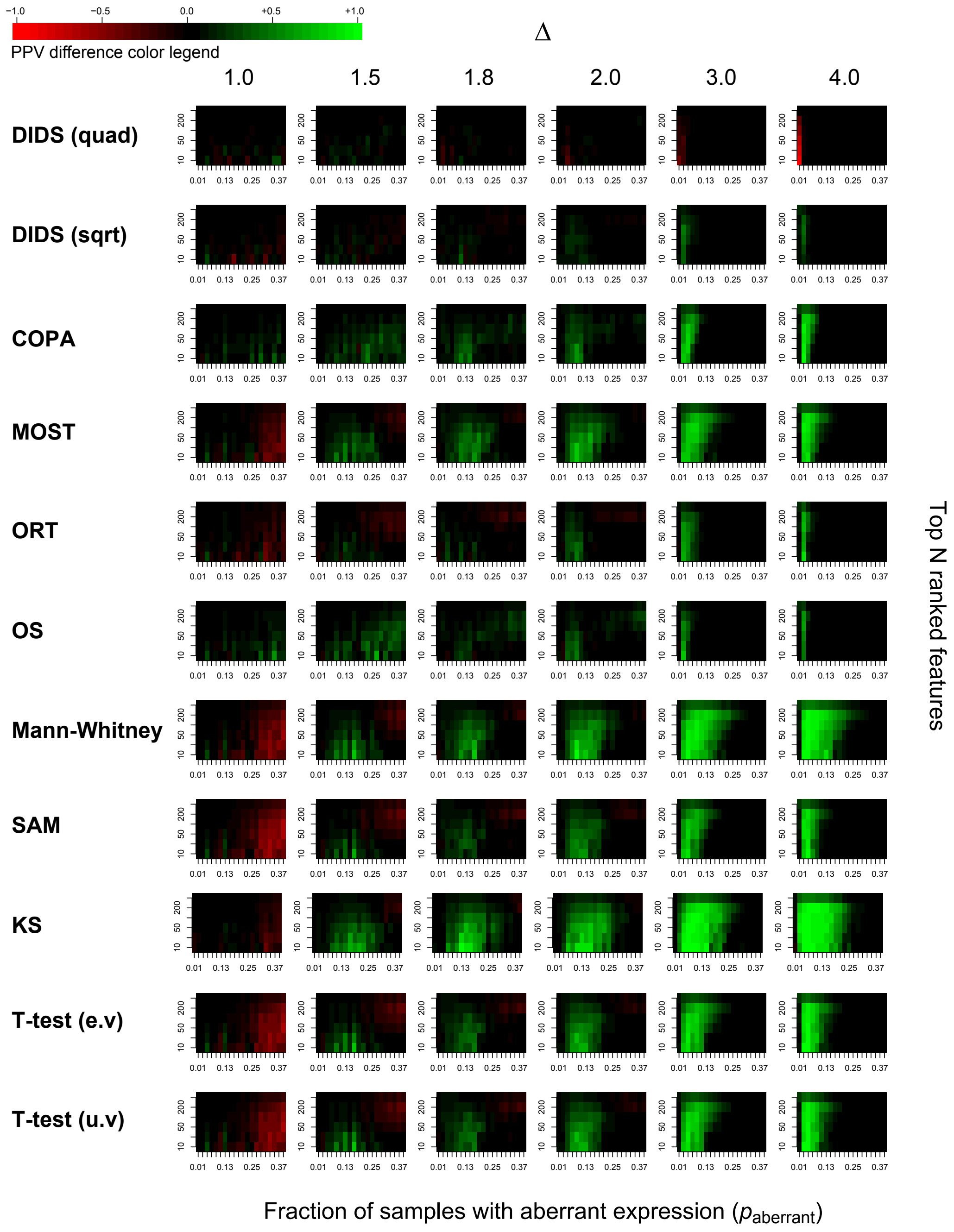

Supplemental Figure 13: The difference in PPV between DIDS and all other tested approaches for the scenario ($n_1 = 100; n_2 = 100$). The methods are represented in the rows and the different values of Δ in the columns. For each combination of a method and a value of Δ, the PPV differences for DIDS and the method represented in the column are depicted as a function of p_{aberrant} (horizontal axis) and α (vertical axis). Shades of green (red) represent settings where DIDS achieves a higher (lower) PPV than the other methods.

Supplemental Table 1: Three-way ANOVA sum of squares table (10 controls versus 10 cases) Sum of squares are listed for a full factorial design with all double interactions. 'A' represents the test used, 'B' the difference between cases and controls (i.e. Δ) , 'C' the percentage of aberrant cases (i.e. p_{aberrant}), and 'D' the p-value cut-off (i.e. α).

	**Df**	**Sum Sq**	**Mean Sq**	**F value**	**Pr(>F)**
**A**	11	39.6	3.6	2889.4	0
**B**	5	47.7	9.5	7660.3	0
**C**	19	32.2	1.7	1361.2	0
**D**	11	95.7	8.7	6994.1	0
**A:B**	55	51.7	0.9	755.9	0
**A:C**	209	16.5	0.1	63.3	0
**A:D**	121	13.9	0.1	92.3	0
**B:C**	95	21.5	0.2	181.7	0
**B:D**	55	24.9	0.5	363.5	0
**C:D**	209	24.4	0.1	93.8	0
**A:B:C**	1045	19.5	0.0	15.0	0
**A:B:D**	605	14.4	0.0	19.1	0
**A:C:D**	2299	8.9	0.0	3.1	0
**B:C:D**	1045	8.8	0.0	6.8	0
**Residuals**	11495	14.3	0.0	NA	NA

Supplemental Table 2: Three-way ANOVA sum of squares table (25 controls versus 95 cases)
Sum of squares are listed for a full factorial design with all double interactions. 'A' represents the test used, 'B' the difference between cases and controls (i.e. Δ) , 'C' the percentage of aberrant cases (i.e. p_{aberrant}), and 'D' the p-value cut-off (i.e. α).

	**Df**	**Sum Sq**	**Mean Sq**	**F value**	**Pr(>F)**
**A**	11	147.7	13.4	3281.2	0.0
**B**	5	462.3	92.5	22589.5	0.0
**C**	19	224.8	11.8	2890.5	0.0
**D**	11	247.0	22.5	5485.7	0.0
**A:B**	55	151.4	2.8	672.8	0.0
**A:C**	209	92.0	0.4	107.6	0.0
**A:D**	121	7.5	0.1	15.1	0.0
**B:C**	95	94.0	1.0	241.8	0.0
**B:D**	55	28.2	0.5	125.4	0.0
**C:D**	209	39.2	0.2	45.8	0.0
**A:B:C**	1045	80.8	0.1	18.9	0.0
**A:B:D**	605	14.9	0.0	6.0	0.0
**A:C:D**	2299	27.8	0.0	3.0	0.0
**B:C:D**	1045	11.6	0.0	2.7	0.0
**Residuals**	11495	47.0	0.0	NA	NA

Supplemental Table 3: Three-way ANOVA sum of squares table (50 controls versus 100 cases) Sum of squares are listed for a full factorial design with all double interactions. 'A' represents the test used, 'B' the difference between cases and controls (i.e. Δ) , 'C' the percentage of aberrant cases (i.e. p_{aberrant}), and 'D' the p-value cut-off (i.e. α).

	**Df**	**Sum Sq**	**Mean Sq**	**F value**	**Pr(>F)**
**A**	11	105.0	9.5	2541.2	0.00E+000
**B**	5	731.2	146.2	38941.4	0.00E+000
**C**	19	481.3	25.3	6746.0	0.00E+000
**D**	11	284.3	25.8	6882.5	0.00E+000
**A:B**	55	133.8	2.4	647.8	0.00E+000
**A:C**	209	93.6	0.4	119.3	0.00E+000
**A:D**	121	6.7	0.1	14.7	1.04E-269
**B:C**	95	139.8	1.5	391.9	0.00E+000
**B:D**	55	22.7	0.4	110.0	0.00E+000
**C:D**	209	38.0	0.2	48.4	0.00E+000
**A:B:C**	1045	65.9	0.1	16.8	0.00E+000
**A:B:D**	605	9.6	0.0	4.2	1.90E-206
**A:C:D**	2299	26.6	0.0	3.1	0.00E+000
**B:C:D**	1045	30.1	0.0	7.7	0.00E+000
**Residuals**	11495	43.2	0.0	NA	NA

Supplemental Table 4: Three-way ANOVA sum of squares table (100 controls versus 100 cases)

Sum of squares are listed for a full factorial design with all double interactions. 'A' represents the test used, 'B' the difference between cases and controls (i.e. Δ) , 'C' the percentage of aberrant cases (i.e. p_{aberrant}), and 'D' the p-value cut-off (i.e. α).

	Df	Sum Sq	Mean Sq	F value	Pr(>F)
**A**	11	54.3	4.9	1542.2	0.00E+00
**B**	5	867.3	173.5	54141.1	0.00E+00
**C**	19	749.4	39.4	12311.5	0.00E+00
**D**	11	283.2	25.7	8036.3	0.00E+00
**A:B**	55	118.8	2.2	674.0	0.00E+00
**A:C**	209	97.6	0.5	145.8	0.00E+00
**A:D**	121	6.3	0.1	16.3	4.49E-302
**B:C**	95	154.5	1.6	507.5	0.00E+00
**B:D**	55	25.4	0.5	144.1	0.00E+00
**C:D**	209	31.5	0.2	47.0	0.00E+00
**A:B:C**	1045	54.0	0.1	16.1	0.00E+00
**A:B:D**	605	6.4	0.0	3.3	1.01E-130
**A:C:D**	2299	22.1	0.0	3.0	0.00E+00
**B:C:D**	1045	52.2	0.0	15.6	0.00E+00
**Residuals**	11495	36.8	0.0	NA	NA
